# Differential Genotypic Effects of HSV-1 Infection on Cognitive Function and Pathology in the 5xFAD Mouse Model

**DOI:** 10.3390/cells15201841

**Published:** 2026-10-10

**Authors:** Kerry Lin, Emily Gu, Sydney Ackerman, Xisheng Wang, Amelie F. Huener, Ibrahim Abou-Seada, Alison Li, Kathy R. Magnusson, Ling Jin

**Affiliations:** 1 Carlson College of Veterinary Medicine, Oregon State University, Corvallis, OR 97331, USA; linker@oregonstate.edu (K.L.); gue@oregonstate.edu (E.G.); wangxis@oregonstate.edu (X.W.);; 2 University of Veterinary Medicine Hannover, 30559 Hannover, Germany; afhuener@t-online.de; 3 Linus Pauling Institute, Oregon State University, Corvallis, OR 97331, USA; abouseai@oregonstate.edu

**Keywords:** HSV-1, heterozygous 5XFAD mice (Het), C57BL/6 (WT), cognitive behavior, spatial learning and memory, amyloid beta or amyloid plaques, IL-1β, TNF-α, real-time PCR, reactive astrocytes, HSV-1 latency, HSV-1 reactivation

## Abstract

**Highlights:**

A recent infectious theory proposes that pathogens, such as HSV-1, could be the root cause of Alzheimer’s disease (AD). We hypothesize that the persistent HSV-1 genome or viral proteins in the infected brain may cause cognitive decline and AD-like pathology. We investigated this hypothesis in two animal models: heterozygous 5XFAD mice (Het), carrying five AD-linked mutations on a C57BL/6 background, and wild-type littermates (WT). Although increased amyloid plaque accumulation was observed only in infected Het mouse brains, long-term memory was affected in both infected Het and WT mice. HSV-1 DNA and viral protein were detectable in both infected Het and WT mice at 90 dpi. A 50–75% reactivation rate was observed in infected brain tissues collected at 90 dpi, including the cerebrum, hippocampus, cerebellum, and brainstem. This study revealed that an aberrant HSV-1 latency in the brain may increase amyloid plaque accumulation and inflammation, which could contribute to cognitive decline and AD-related pathology.

**What are the main findings?**

- HSV-1 reduced learning in latently infected mice regardless of genotype.
- HSV-1 infection increased amyloid beta accumulation in the hippocampus of Het mice.
- Inflammatory cytokines were increased in the hippocampus in Het and WT mice latently infected with HSV-1.
- Reactive astrocytes were increased in the CA1 region in WT mice latently infected with HSV-1.
- Around 50% to 75% reactivation via explant was detected in many regions of the brain collected at 90 dpi. The degree of HSV-1 reactivation in the brain was relatively lower than that in the trigeminal ganglia.

**What are the implications of the main findings?**

- HSV-1 may become aberrantly latent in the brain with persistence of both viral genome and viral protein, unlike conventional latency in the trigeminal ganglia.
- Aberrant HSV-1 latency in the brain may trigger neuroinflammatory responses and contribute to cognitive decline and brain pathology.

**Abstract:**

Alzheimer’s disease (AD) involves several different etiopathogenic mechanisms. A recent infectious theory proposes that pathogens, such as HSV-1, could be the root cause of AD. In this study, we tested this infectious theory in an HSV-1 mouse model using heterozygous 5XFAD mice (Het) carrying 5 AD-linked mutations on a C57BL/6 background and wild-type littermates (WT). We hypothesized that HSV-1 could persist in the latently infected brain and that multiple heat stresses could cause greater HSV-1 persistence, cognitive decline and AD-like pathology. Infected (HSV+) mice and non-infected (HSV-) mice with (HS) or without (NHS) heat stress at 30 and 60 dpi were examined 3 days post-heat stress for each genotype. Impairments in long-term learning were observed in infected mice after 30 dpi, regardless of genotype or heat stress. Heat stress alone impaired memory at 30 dpi across genotypes and cognitive flexibility at 60 dpi in Het mice, but this appeared to be due to the recency of the stress. When another cohort of mice with or without 1–3 heat stresses at 30, 45, and 60 dpi was examined 15 days post-heat stress, long-term learning decline was observed in all latently infected mice, regardless of genotype or heat stress. There was significantly more amyloid plaque staining in the hippocampal CA1 region in Het/HSV+/NHS mice than in Het/HSV-/NHS mice. No amyloid plaque staining was observed in WT mice, regardless of HSV-1 infection or stress. Interestingly, TNF-α transcription was higher in the Het/HSV+ mice than in Het/HSV- mice in the hippocampal region. However, IL-1β transcription was found to be significantly higher in the hippocampal region in WT/HSV+ mice with or without stress than in WT/HSV-/NHS mice. There were more active astrocytes in the CA1 region of the hippocampus of WT/HSV+ mice than in WT/HSV- mice. Active astrocyte staining did not differ significantly between infected and uninfected Het mice. Viral DNA was detected in the brains of recovered Het or WT mice at 90 dpi. HSV-1 reactivation from latency was observed in 50–75% of the explants of the cerebrum and cerebellum at 33 dpi. Therefore, a combination of amyloid plaque accumulation, neuroinflammation and viral persistence may underlie the cognitive declines observed in infected Het and WT mice.

## 1. Introduction

Alzheimer’s disease (AD) is the leading cause of dementia among older people worldwide [1]. Clinically, AD is characterized by progressive cognitive impairments, such as declines in memory, language, visuospatial tasks, and sometimes visual impairments [2,3]. Pathologically, the disease is recognized by the presence of amyloid plaques, neurofibrillary tangles (NFTs), neuronal and synaptic loss, dystrophic neurites, reactive astrocytes, and activated microglia. The amyloid hypothesis suggests that the processes governing the production, accumulation, or disposal of beta-amyloid are the primary cause of AD [1]. Familial, early-onset AD is strongly associated with mutations in genes, such as presenilin (PSEN) and amyloid precursor protein (APP) [4], which tend to produce more amyloid in transgenic mice than in control mice [5,6]. However, less than 5% of Alzheimer’s cases are caused by single-gene mutations in the APP, PSEN1, or PSEN2 genes [7]. The majority of late-onset AD cases are influenced by common gene variants, such as the *APOE ε4* allele, lifestyle factors and unknown factors [7,8,9]. An infectious hypothesis has emerged recently and proposes that pathogens are the drivers of AD [1,10]. This theory is based on evidence that some pathogens, such as genomes of herpesviruses and certain bacterial species, are often found in amyloid plaques from the brains of AD patients [11,12,13,14]. In addition, it has been reported that amyloid beta (Aβ) and tau may be the antiviral products of normal responses to infection, which serve to sequester the threat to the CNS [15,16,17]. Therefore, pathogens, such as HSV-1, could be a potential driver of AD pathologies, such as amyloid plaques.

Serology data indicate that HSV-1 infection is widespread in humans, with seropositivity reported at 45–98% worldwide and 57–85% in the United States [18]. Initial infection with HSV-1 is usually asymptomatic, although there may be minor local vesicular lesions on mucosal surfaces or the cornea. During primary infection, HSV-1 enters nerve endings near the infection site, then travels along nerve axons via retrograde transportation to the trigeminal ganglia (TG), dorsal root ganglia, or brainstem, where it establishes a life-long latent infection. HSV-1 reactivations are responsible for cold sores and recurrent corneal disease following stress or immune suppression. During HSV-1 latency, the viral genome is mostly known to persist in the TG and the brainstem [19]. The AD infectious theory comes from post-mortem studies showing that a high level of HSV-1 DNA was detected in amyloid plaques from the brain tissues of AD patients [1,20,21,22]. It has been reported that reactivation of HSV-1 increases the risk of AD [21,23,24,25]. Anti-herpetic medication can reduce the risk of dementia in patients with HSV-1 infections [26]. Growing numbers of publications demonstrate that pathological hallmarks of AD can be detected in the brain during HSV-1 infection or reactivation of HSV-1 infection [24,27,28]. Recently, Tau phosphorylation in response to HSV-1 has been linked to AD-like pathology in the mouse brain [29]. It is well established that recurrent infections are resolved following the end of a stressful stimulus, and peripheral tissues can recover from tissue damage after each reactivation episode. It is still unclear what role HSV-1 latency or reactivation plays in the pathogenesis of AD-like diseases.

It is not known why HSV-1 DNA is present in the AD plaques, or why more amyloid beta and *p*-tau accumulate in the HSV-1-infected mouse brain. Our working hypothesis is that components of HSV-1, such as DNA and viral proteins, are not fully removed from infected neurons and can trigger AD-related pathology and cognitive decline. Heat stress can trigger HSV-1 reactivation from latency [30]. Thus, we also hypothesized that multiple heat stresses would trigger more viral replication and that more viral components would accumulate in the HSV-1 latently infected brain and cause more AD-related pathology. To test this hypothesis, the differences between HSV-1 latently infected animals with or without heat stress were investigated in two mouse lines: heterozygous 5xFAD on a C57BL/6J background (Het) and wild-type C57BL/6J littermates (WT). The 5xFAD mouse, an amyloid overexpression model, carries five AD-linked mutations: the Swedish (K670N/M671L), Florida (I716V), and London (V717I) mutations in APP, and the M146L and L286V mutations in PSEN1, which encodes one subunit of γ-secretase [31]. Heterozygous 5XFAD (Het) mice are commonly used as an Alzheimer’s disease model characterized by rapid, age-related amyloid plaque accumulation, robust neuroinflammation, and significant memory deficits starting at 4–6 months [32]. This study investigated the effects of HSV-1 latency on spatial long-term learning, memory and cognitive flexibility, and AD-associated pathology in Het and WT mice that experienced heat stress and those that did not.

## 2. Materials and Methods

### 2.1. Animals: The 5xFAD Mouse Model Used for This Research Project, B6.Cg

Tg(APPSwFlLon,PSEN1*M146L*L286V)6799Vas/Mmjax, RRID:MMRRC_034848-JAX, was obtained from the Mutant Mouse Resource and Research Center (MMRRC) at The Jackson Laboratory, an NIH-funded strain repository, and was donated to the MMRRC by Robert Vassar, Ph.D., Northwestern University. The 5XFAD mice were developed by Oakley et al. [31] and co-express human APP and presenilin 1 with five familial AD mutations (APP K670N/M671L + I716V + V717I and PS1 M146L + L286V). Heterozygous mice and wild-type (WT) littermates were obtained by crossing 5XFAD hemizygous males on a congenic C57BL/6 genetic background with wild type C57BL/6J (WT) females (The Jackson Laboratory). Genotyping was performed by PCR with total DNA from ear punch tissue. The sequences of the primers used are reported on the website: https://www.jax.org/Protocol?stockNumber=008730&protocolID=31769 (accessed on 15 April 2022) (Table 1). Mice were housed in the Laboratory Animal Resources Center at Oregon State University and maintained in a standard 12 h light/12 h dark cycle environment. All animal procedures were approved by the Oregon State University Institutional Animal Care and Use Committee and conformed to recommendations of the American Veterinary Association Panel on Euthanasia (IACUC-2021-0194). Mice were randomly assigned to groups, and researchers performing assays were blind to the group assignments.

### 2.2. Cells and Viruses

Rabbit skin (RS) cells used for virus titration and Vero cells used for viral stock preparation were cultured in Dulbecco’s modified Eagle’s medium (DMEM) supplemented with 10% fetal bovine serum (FBS) (Invitrogen,, Carlsbad, CA, USA), penicillin (100 U/mL), streptomycin (100 g/mL) (Sigma–Aldrich, Inc. St. Louis, MO, USA), and gentamicin (10 g/mL) (Invitrogen, Carlsbad, CA, US) at 37 °C with 5% CO_2_ in a humidified incubator. The GFP-HSV-1 [33] was propagated in Vero cells and used in the infection. The quantity of viruses used in the experiments was determined by standard plaque assay using RS cells as reported previously [34].

### 2.3. Mice Infection and Heat Stress

At 7–8 weeks of age, mice were infected with GFP-HSV-1 virus. Each eye was inoculated twice with 1 × 10^5^ PFU of GFP-HSV-1 virus in 10 µL, with at least 5 min between the 2 inoculations (total = 2 × 10^5^ PFU/eye). Infection was monitored by eye swabbing at 1, 3, 7, and 10 days post-infection (dpi). Each cotton swab was inserted into 0.5 mL of PBS buffer and analyzed by plaque assays. Mice were stressed in a 42 °C water bath cage for 10 min each. Timing post-infection and the number of stresses differed between the 2 studies. Mice (3–4 per cage) were placed in a standard mouse cage filled with 1.5 inches of 42 °C water; the lidded cage was placed inside the 42 °C water bath. Mice were kept in the cage for 10 min.

Study 1: Four groups per genotype (3–6 mice per group; ± infected X ± heat stressed) were assessed in behavioral tasks. Detailed animal infection status and gender are listed in Table S1. Heat stress was applied once at 30 dpi (HS1) and again at 60 dpi (HS2); behavioral tests were performed 3 days after each heat stress.

Study 2: To rule out active viral replication influencing cognitive function (during behavior test) in mice tested 3 days post-infection, we assessed a second set of mice (3–4 mice per group; 2 genotypes X ± infected X 0–3 heat stresses). The detailed animal infection status and gender are listed in Table S2. Heat stress was applied once at 30 dpi (HS1), twice at 30 dpi and 45 dpi (HS2), or three times at 30 dpi, 45 dpi, and 60 dpi (HS3). Cognitive function was assessed using the water maze 15 days after the final 60 dpi heat stress to ensure the behavior analysis was performed at least 2 weeks after recovery and during HSV-1 latency.

### 2.4. Behavioral Testing

Spatial long-term learning and memory, cognitive flexibility, and associative memory (cued control task) were tested using the Morris water maze. The maze consisted of a white circular pool (122 cm in diameter and 51 cm in height) filled with water (20 ± 2 °C) and made opaque by a white, nontoxic pigment. A platform (9 cm in diameter) that was not visible from the water surface was positioned 1 cm below the water surface in the NW quadrant. For the first 2 days, mice were acclimated to the water maze (swimming 1 min, 30 s staying on platform), followed by 3 days of testing for spatial learning and memory, 1 day of reversal training to test cognitive flexibility, and 1 day of associative memory testing (cued control task). Spatial learning and cognitive flexibility testing consisted of four place trials with an inter-trial interval of 20 min each day. Probe trials (no platform) to test memory and flexibility were performed before the first learning and memory trial and ~22 h after the last trials on Days 2 and 3 of learning and memory testing and after reversal training. The control task consisted of 6 trials with the platform visible and in different positions each trial. For all behavioral testing, the animal’s movements in the water maze were tracked and analyzed with the AnyMaze system (San Diego Instruments, San Diego, CA, USA) as previously described [35].

### 2.5. Brain Sectioning

Following behavioral tests, mice were euthanized by exposure to CO_2_. Brains were harvested and then divided into two halves in the sagittal plane. One half was sectioned for amyloid plaque staining or immunofluorescent staining; the other half was used in DNA and RNA analysis. Chilled gelled slides (0.5% gelatin solution) were used for collecting brain tissue sections. Horizontal brain sections of 10 μm thickness, from representatives of each group, were sectioned using a cryostat (Leitz, Boston, MA, USA) and placed on each slide and stored at −80 °C until used.

### 2.6. Amyloid Plaque Staining, FJC Staining and Imaging Analysis

Amyloid plaque staining was performed with Amylo-Glo RTD Amyloid Plaque Stain Reagent (TR-300-AG, Biosensis, Thebarton, Australia) according to the manufacturer’s instructions. Amylo-Glo-stained amyloid plaques were visualized using UV epifluorescent illumination. The stained area and particle numbers were analyzed by ImageJ software. FJC staining was performed to detect degrading neurons according to the manufacturer’s protocol (Fluoro-Jade C staining Kit, Biosensis, Thebarton, South Australia) and stained sections were then stained by ICP27 antibody as described below.

### 2.7. DNA Extraction and PCR Genotyping

Total tissue DNA was isolated from ear notches using the EZNA Tissue DNA extraction kit (Omega Bio-tek Inc. Norcross, GA, USA). 5XFAD PCR was run with 1.30X Kapa 2G HS buffer, 2.60 mM MgCl2, 0.26mM dNTP, 0.50 µM of common forward primer, 0.50 µM of mutant reverse primer, and wild-type reverse primer, 6.50% glycerol and 0.03U/ul Kapa 2G HS taq polymerase. The reaction was run at 94 °C for 2 min, then 10 touchdown cycles at 94 °C for 30 s, 65 °C for 30 s with 0.5 °C per cycle decrease, 68 °C for 30 s; then 30 cycles of 94 °C for 30 s, 60 °C for 45 s, and 72 °C for 30 s, followed by 72 °C for 5 min and a final hold at 20 °C. The PCR products were run in a 1.5% agarose gel with 1X SYBR green.

### 2.8. HSV-1 Real-Time PCR and PCR

Brain tissues were homogenized in 100 µL of PBS with silica beads and total tissue DNA was isolated as described above. The viral genome in different tissues was analyzed with Taqman real-time PCR with LAT- and gD-specific primers (Table 1) and Taqman probes [36]. The relative copy numbers of gD and LAT DNA were calculated using standard curves generated from a plasmid containing the PCR products, which are shown in Figure S1A,B. UL36 nested PCR amplification was performed using a 25 μL solution consisting of 2.5 μL amplification buffer (10X PCR buffer with 15 mM MgCl2), 0.4 mM dNTP, 0.4 μM each primer, 1 U Taq (Thermo Fisher Scientific, Eugene, OR, USA), and 20–500 ng DNA sample. The mixture was subjected to 94 °C for 2 min, 35 cycles of 94 °C for 30 s, 55 °C for 30 s, and 72 °C for 30 s, followed by a 5 min elongation reaction at 72 °C after the final cycle. The nested PCR was performed using a nested set primers (Table 1). A 2.5 μL aliquot of the first-run PCR product was included as a template in the second (nested) amplification under the same PCR running conditions. The PCR products were run in a 1.5% agarose gel with 1X SYBR green. The 1 Kb Plus DNA Ladder (Thermo Fisher Scientific, Eugene, OR, USA) served as size markers in gel electrophoresis.

### 2.9. RNA Extraction and Reverse Transcriptase-Real-Time PCR

Tissue samples of ~100 mg were homogenized in 100 µL of PBS with silica beads. Total RNA was extracted from mouse tissue with Trizol according to the manufacturer’s instructions (Invitrogen). The cDNA was synthesized with the qScript cDNA Synthesis Kit (Quantabio, Beverly, MA, USA), which was then amplified by SYBR real-time PCR Master Mix (Thermo Fisher Scientific) with primers specific to mouse TNF-α, IL-1β, and β-actin (Table 1) as described before [37,38]. β-actin is a structural cytoplasmic protein involved in cell motility and cytoskeleton maintenance, and its gene expression is widely used as an internal housekeeping gene in RT-PCR analysis [38]. The amount of cDNA used from each sample in RT-qPCR was normalized to β-actin transcription, as shown in Figure S2. A standard curve was made for each gene by cloning the PCR products of TNF-α (Figure S1C) and IL-1β (Figure S1D) into a pCR2.1 TOPO vector (Invitrogen). The Ct value was converted to gene copy number per µg based on the standard curves shown in Supplementary Figure S1. Therefore, the results shown in the figure represent the absolute transcript copy number per microgram of total RNA from each tissue sample.

### 2.10. Indirect Immunofluorescence Assay (IFA) and FJC-ICP27 Co-Staining

Mouse frozen sections were fixed in acetone for 10 min at −20 °C and air dried for 20 min before staining. Slides were first blocked with 10% horse serum blocking buffer for 30 min at room temperature, then incubated with primary antibodies, HSV-ICP27 monoclonal antibody (H1113) (Virusys Monoclonal Antibodies, Milford, MA, USA) at 1:200 or Glial Fibrillary Acidic protein (GFAP) rabbit polyclonal antibody (R-1374-50, Biosensis, Thebarton SA 5031, Australia) at 1:2000, in a humidified chamber at 4 °C overnight. A secondary goat anti-rabbit IgG antibody conjugated with tetramethylrhodamine isothiocyanate (TRITC) (Cat No. 4040-03), or a secondary goat anti-mouse antibody conjugated with FITC (cat no. 1070-02) from Southern Biotech was applied to slides at a dilution of 1:2000 and incubated in a humidified chamber at room temperature for 1 h. Slides were washed gently 3–4 times in PBS containing 0.01% Tween 20 at room temperature for 5 min before and after application of the secondary antibody. Slides were then covered with Vectashield® mounting media containing DAPI (Vector® Laboratories, Newark, CA, USA) before application of a coverslip and examined under a fluorescent microscope.

FJC-ICP27 staining: Tissue sections were stained first with FJC reagent following the manufacturer’s instructions. The tissue sections were then fixed again in 4% paraformaldehyde for 20 min, washed twice with PBS for 5 min each, and permeabilized in 0.1% Triton X-100 for 10 min, followed by two PBS washes. The tissue sections were blocked in 2% BSA buffer and stained as described above with the ICP27 monoclonal antibody first at room temperature for 1 h, washed gently 3 times in PBS containing 0.01% Tween 20 at room temperature for 5 min before and after application of the secondary goat anti-mouse antibody conjugated with TRITC (Red) from Southern Biotech, Birmingham, AL, USA (Cat No. 1070-03). The secondary antibody was incubated for 0.75–1 h before washing. The slides were viewed directly under the fluorescence microscope.

### 2.11. Tissue Explant

To determine whether HSV-1 becomes latent in the mouse brain, the frontal cortex (FC), caudal cortex (Cx), hippocampus (HP), cerebellum (C), brainstems (B), and TG were each explanted at 33 dpi and cultured in 1.5 mL MEM in a 12-well plate as we described previously [33,39]. Infectious virus from the explant was monitored daily for 10 days in a 100 µL aliquot of the explant culture, which was then used to infect RS cells in a 96-well plate. The cytopathic effect (CPE) or GPF expression from HSV-1 infection was monitored for 5 days to detect reactivated viruses in each brain tissue explant culture.

### 2.12. Statistics

Behavioral test results were analyzed by repeated-measures ANOVA (Genotype X Infection X Heat Stress(es) X Trial (repeated measure)) and Fisher’s LSD post hoc analysis of main effects with Jamovi version 2.2.5 or Prism 11.1. All other statistical analyses were performed using GraphPad Prism, version 11.0.1 for Windows (GraphPad Software, San Diego, CA, USA). The differences in beta amyloid expression and gene expression were analyzed by unpaired *t*-tests.

## 3. Results

### 3.1. HSV-1 Infection Can Become Latent in Heterozygous 5xFAD (Het) Mice and Reactivate Following Heat Stress

**Figure 1:**
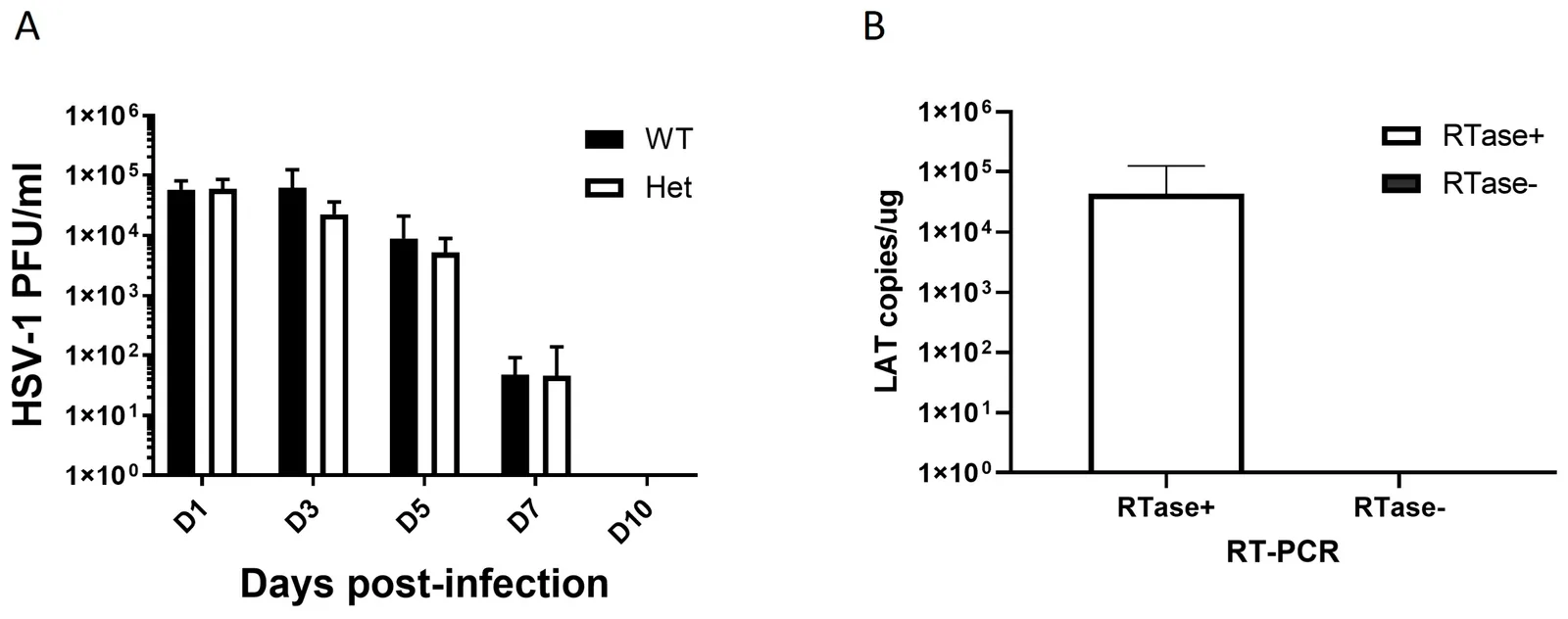
Virus shedding in the tears during acute infection and detection of LAT in Het TG at 30 dpi. (**A**): Virus titration of mouse tear swabs from HSV-1-infected Het and WT mice. Both eyes were infected with 2 × 10^5^ PFU HSV-1 McKrae/eye. Tear swabs were collected on 1, 3, 5, 7, and 10 days post-infection (dpi) from 5 mice per group. WT: Wild-type, Het: 5 × FAD heterozygotes. (**B**): Detection of HSV-1 latency-associated transcript (LAT) by reverse transcription real-time PCR with total RNA of TG from HSV-1-infected Het mice at 30 days post-infection. TGs were taken from three HSV-1-infected Het mice. RTase+: cDNA synthesis with reverse transcriptase, RTase-: cDNA synthesis without reverse transcriptase. N = 3.

**Figure 2:**
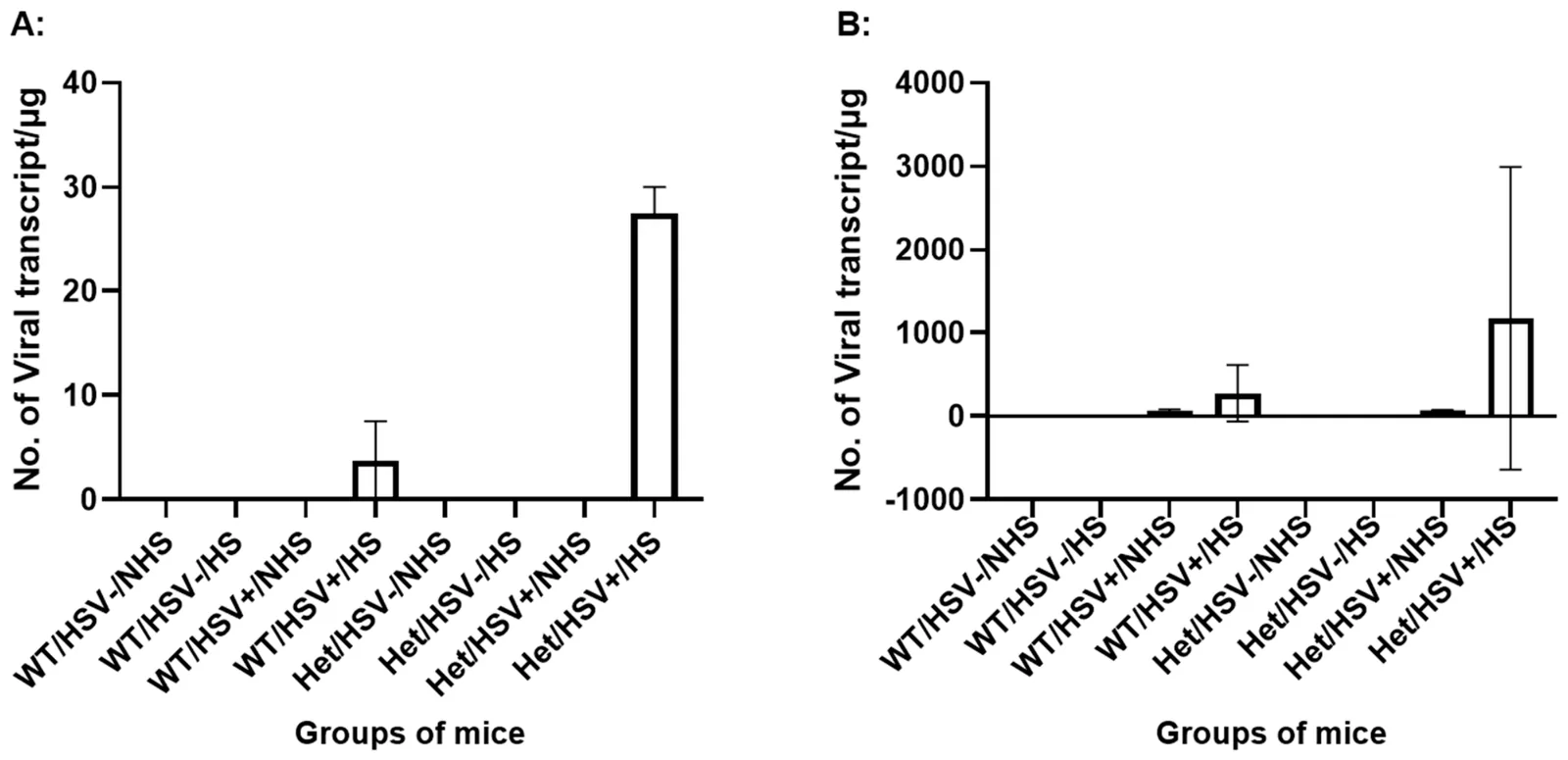
Quantification of HSV-1 LAT (**A**) and gD (**B**) in the mouse frontal lobe 3 days post-heat stress. The x-axis represents groups of mice ± infection with HSV-1 (2 × 10^5^ PFU/eye of HSV-1 McKrae). The y-axis represents the number of LAT or gD copies detected by reverse transcription real-time PCR. NHS: No heat stress. HS: Heat stress on day 30 post-infection. Het: 5XFAD mice. WT: C57BL/6J, wild-type littermate mice. N = 3.

To determine whether HSV-1 infection can become latent in heterozygous 5xFAD (Het) mice [34] and wild-type (WT) C57BL/6 mice littermates, mice were infected at 7–8 weeks of age with ~2×10^5^ PFU GFP-HSV-1/McKrae per eye [33]. Virus shedding was monitored by eye swabbing and titered via a standard plaque assay. Both infected WT and Het mice shed between 1×10^4^ and 1×10^5^ PFU per mL during the first 5 dpi and cleared the infection by 10 dpi (Figure 1A). To prove HSV-1 becomes latent in the TG of infected Het mice, the expression of the latency-associated transcript (LAT), which is the only known viral RNA expressed during latency, was examined by reverse transcription real-time PCR. As shown in Figure 1B, amplification of LAT is only present in the reaction in the presence of reverse transcriptase, which indicates that HSV-1 had become latent in the TG of Het mice (n = 3). Whether HSV-1 reactivation can be induced by heat stress in Het mice was previously unknown. To demonstrate HSV-1 reactivation in the brains of mice following heat stress, viral gene transcription, a feature of lytic infection, was examined in groups of both Het and WT mice (n = 3) subjected to heat stress (HS) or not (NHS) at 30 dpi. HSV-1 late gene gD transcription and HSV-1 LAT transcription, which is also expressed during lytic infection, were quantified by reverse-transcription real-time PCR using total RNA from the frontal lobes. As shown in Figure 2, viral gene transcription of LAT and gD was only detectable 3 days post-heat stress in the frontal lobes of HSV-1-infected (HSV+) and stressed mice. This result demonstrated that lytic infection could occur in the brain in both Het and WT mice following heat stress.

### 3.2. There Were Differences in Spatial Memory and Cognitive Flexibility in HSV-1 Latently Infected Mice Tested Within 3 Days Post-Heat Stress

**Figure 3:**
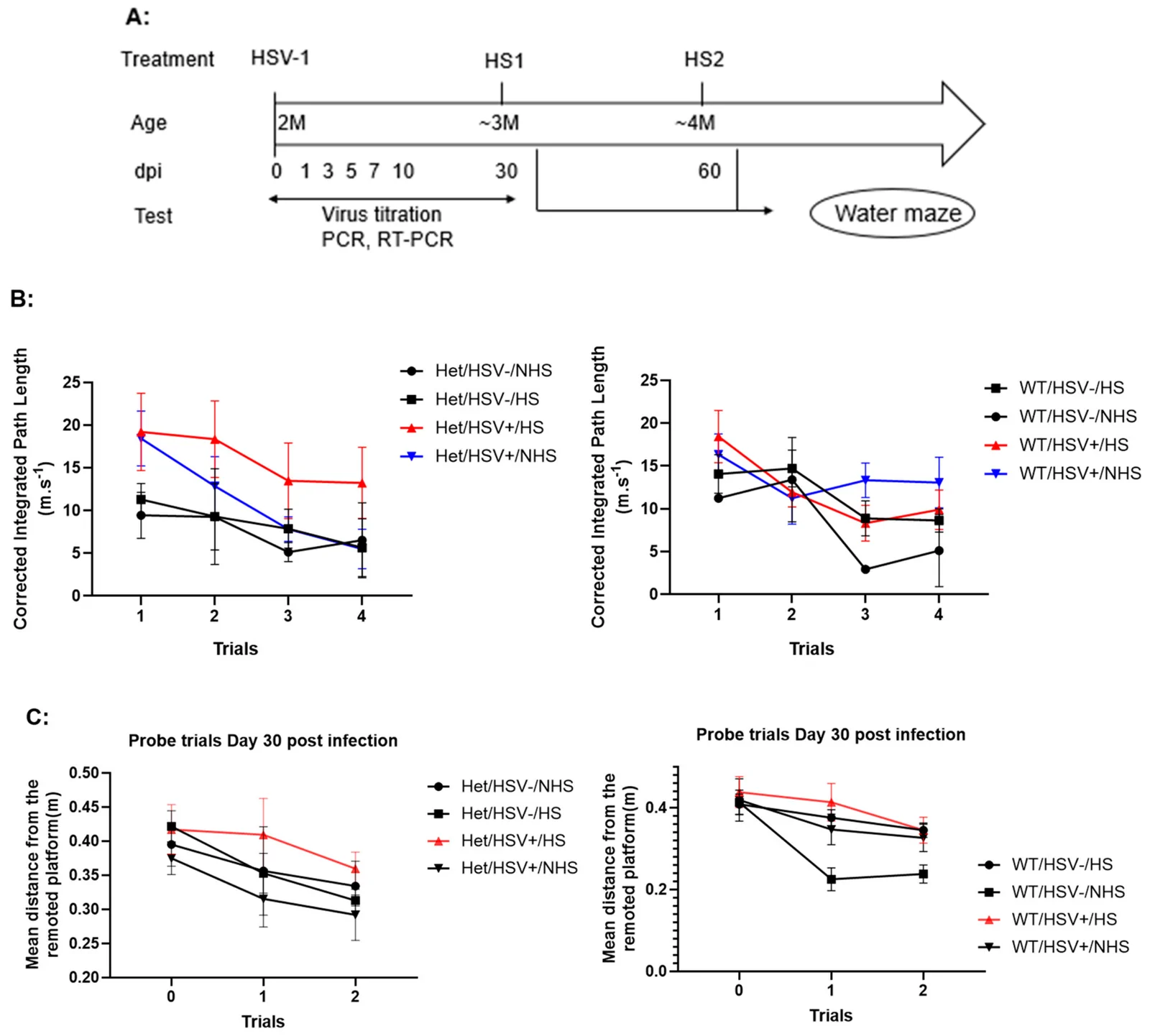
Timeline for 2-heat-stress study and acute behavioral testing. (**A**) Schematic of HSV-1 infection, heat stresses (HS), and memory testing. HS1: Heat stress at 30 days post-infection, HS2: heat stress at 30 and 60 dpi. (**B**) Spatial long-term learning and memory (blocks of 3 place trials) testing in Het (Left) and WT (Right) mice with (HS) or without (NHS) heat stress. (**C**) Probe trials for memory in Het (left) and WT (right) mice with (HS) or without (NHS) heat stress. Mice were stressed once on day 30 post-infection and tested in a Morris water maze 3 days post-stress (42 °C; 10 min). Means ± SEM. ANOVA and Fisher’s LSD post hoc tests. N = 3–6.

To understand whether HSV-1 latency in mice experiencing heat stress or no heat stress has any effect on cognitive function, infected and non-infected mice with or without heat stress at 30 and 60 dpi were examined 3 days post-heat stress for each genotype (Figure 3A). Spatial learning and memory, cognitive flexibility, and associative memory (cued control task) were examined in the Morris water maze as described previously [35]. Each group, with 3–6 mice per group, was tested twice, 3 days after the heat stresses on day 30 and day 60 post-infection [40]. There was a significant main effect of Infection (F (1,34) = 7.44, *p* = 0.01) and Trial block (F (11,374) = 7.88, *p* < 0.001) on the learning phase of long-term memory after 30 dpi, but no significant effect of Genotype (F (1,34) = 0.313, *p* = 0.579) or Heat stress (F (1,34) = 2.52, *p* = 0.121). One month after viral treatment, the HSV-1-infected mice had significantly higher CIPL scores (more searching away from the platform) than uninfected mice, regardless of genotype or stress (Figure 3B). For the probe trials, there was a significant main effect of Heat stress (F (1,34) = 5.08, *p* = 0.031) and Trial (F (2,68) = 11.23, *p* < 0.001), but no effect of Genotype (F (1,34) = 0.16, *p* = 0.69) or Infection (F (1,34) = 0.14, *p* = 0.87) after the 30 dpi stress (Figure 3C). No difference was observed in cued trials among the different treatment groups in either Het or WT mice stressed only at 30 dpi (Figure S3A), suggesting that all groups had similar motivation, visual acuity, and physical ability.

**Figure 4:**
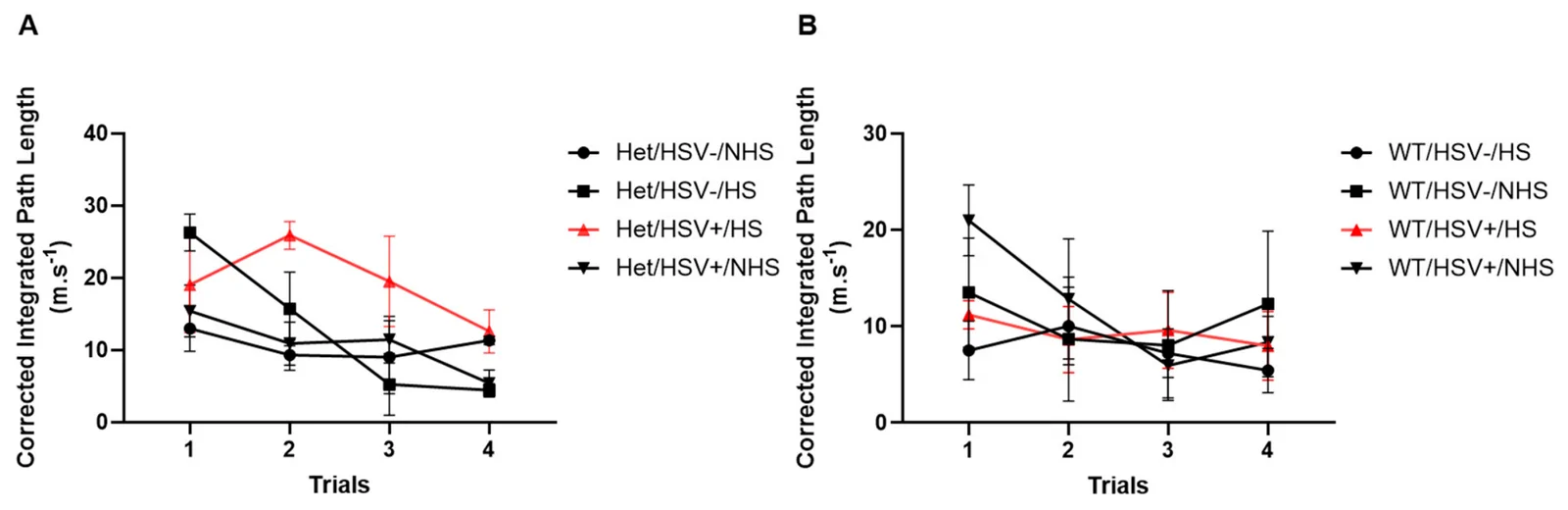
Cognitive flexibility (reversal trials) analysis in Het (**A**) and WT (**B**) mice. Mice were stressed twice, on days 30 and 60 post-infection, and tested 3 days after the second heat stress at 42 °C for 10 min. Means ± SEM. RANOVA and Fisher’s LSD post hoc tests. N = 3–6.

After mice were stressed at both 30 and 60 dpi, no difference in spatial long-term learning and memory was observed regardless of Infection, Stress, or Genotype. However, in reversal trials for cognitive flexibility, there was a significant interaction between Genotype and Reversal trial (F (11,297) = 2.28, *p* = 0.011), so the genotypes were assessed separately. In Het mice, there were significant main effects of Heat stress (F (1,12) = 6.37, *p* = 0.027) and Reversal trial (F (2.28,26.63) = 6.04, *p* = 0.005), but not Infection (F (1,12) = 2.36, *p* = 0.15) in reversal trials (Figure 4A). The heat-stressed Het mice had higher CIPL scores than non-stressed Het mice (Figure 4A). No significant differences in reversal trials were seen between different infection/stress groups in WT mice (Figure 4B). No differences were observed between different infection/stress groups of mice following stress at 60 dpi in cued trials (Figure S3B) or probe trials (Figure S4), regardless of genotype.

### 3.3. There Was a Difference in Spatial Learning Between Uninfected Mice and HSV-1 Latently Infected Mice Tested 2 Weeks Post-Heat Stresses

**Figure 5:**
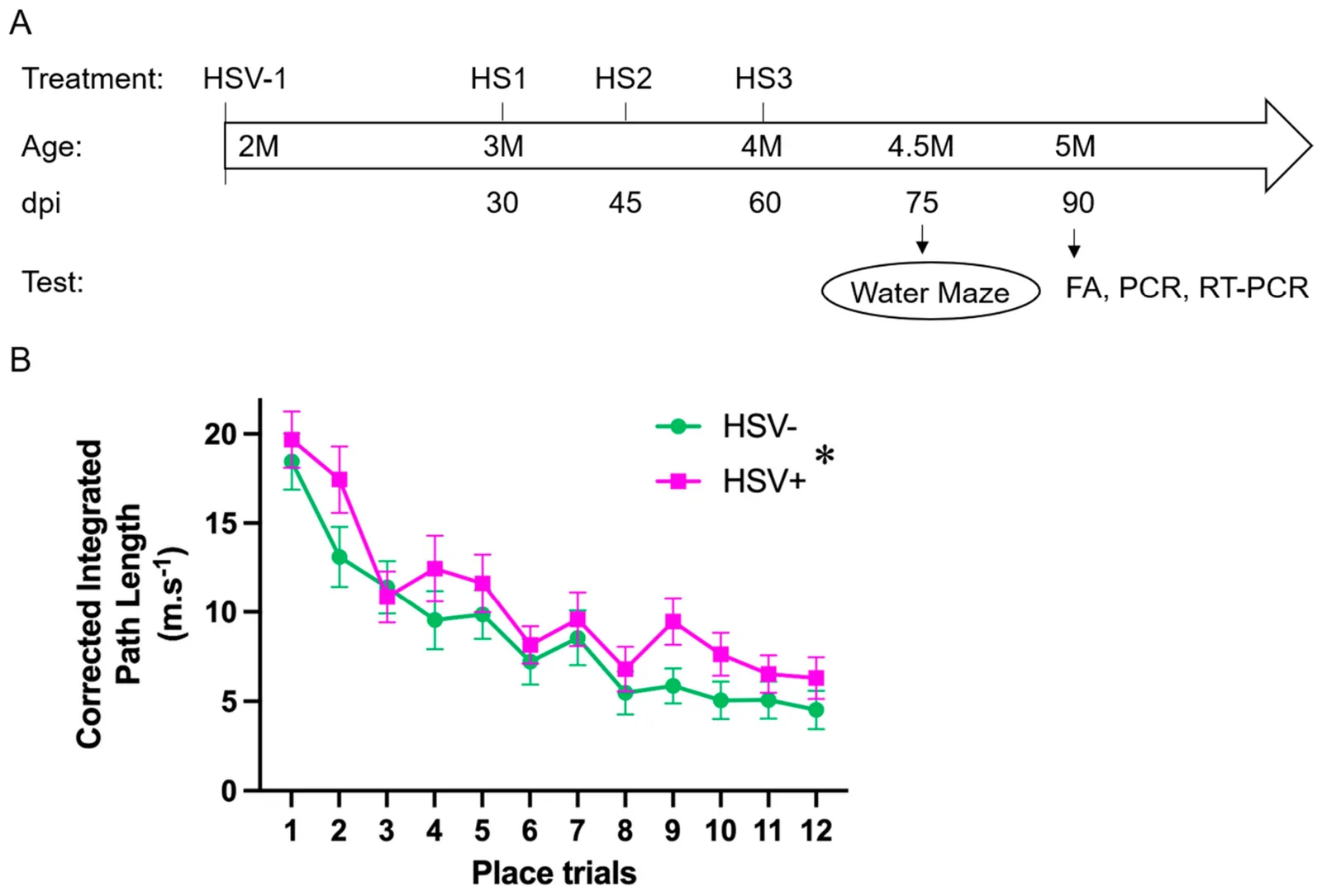
Timeline for 3-heat-stress study and behavioral testing after recovery. (**A**) Schematic of HSV-1 infection, heat stress (HS) and behavioral tests. Het and WT mice were stressed once, twice or 3 times on 30, 45, 60 post-infection, respectively, and tested using a Morris water maze, starting 15 days post-stress (42 °C; 10 min). (**B**) Spatial long-term learning and memory (place trials) testing in HSV+ and HSV- mice. HSV+ mice regardless of stress or genotype had significantly worse performance than HSV- mice in the place trials (*p* = 0.029). Means ± SEM. RANOVA and Fisher’s LSD post hoc tests. HS1: Heat stress at 30 days post-infection, HS2: heat stress at 30 and 45 dpi, HS3: heat stress at 30, 45, and 60 dpi. Pooled N = 27–29 after collapsing data across heat stresses and genotype. *: *p* < 0.01.

Because behavioral analysis was performed 3 days post-heat stress, those mice may still have been experiencing active HSV-1 reactivation during behavior testing. To rule out the possibility of active HSV-1 reactivation from heat stress during the behavior test, additional groups of mice were infected and stressed 0–3 times at 30, 45, and 60 dpi, as indicated in Figure 5A, and the behavior analysis was performed 15 days after the final heat stress to ensure that the differences between mice stressed multiple times and non-stressed mice were all evaluated during the same infection latency period. Due to low Ns for heat stress repetitions in the WT and a lack of genotype effect when numbers of heat stresses were collapsed, genotypes were combined in a repeated-measures (place trials) ANOVA. There was a significant main effect of Infection (F (1,47) = 5.1, *p* = 0.029) and Place trial (F (11,517) = 16.66, *p* < 0.001), but no significant effect of Heat stresses (F (3,47) = 1.128, *p* = 0.347). Infected mice had higher CIPL scores (less searching near the platform) than uninfected mice in place trials for long-term learning and memory, regardless of a variable number of heat stresses or genotype (Figure 5B). No consistent differences in cognitive flexibility or cued trials were observed between different treatment groups in Het or WT mice with or without heat stress.

### 3.4. There Were Significantly More Amyloid Plaques in HSV-1-Infected Het Mice than Uninfected Het Mice in CA1 Region of Hippocampus

**Figure 6:**
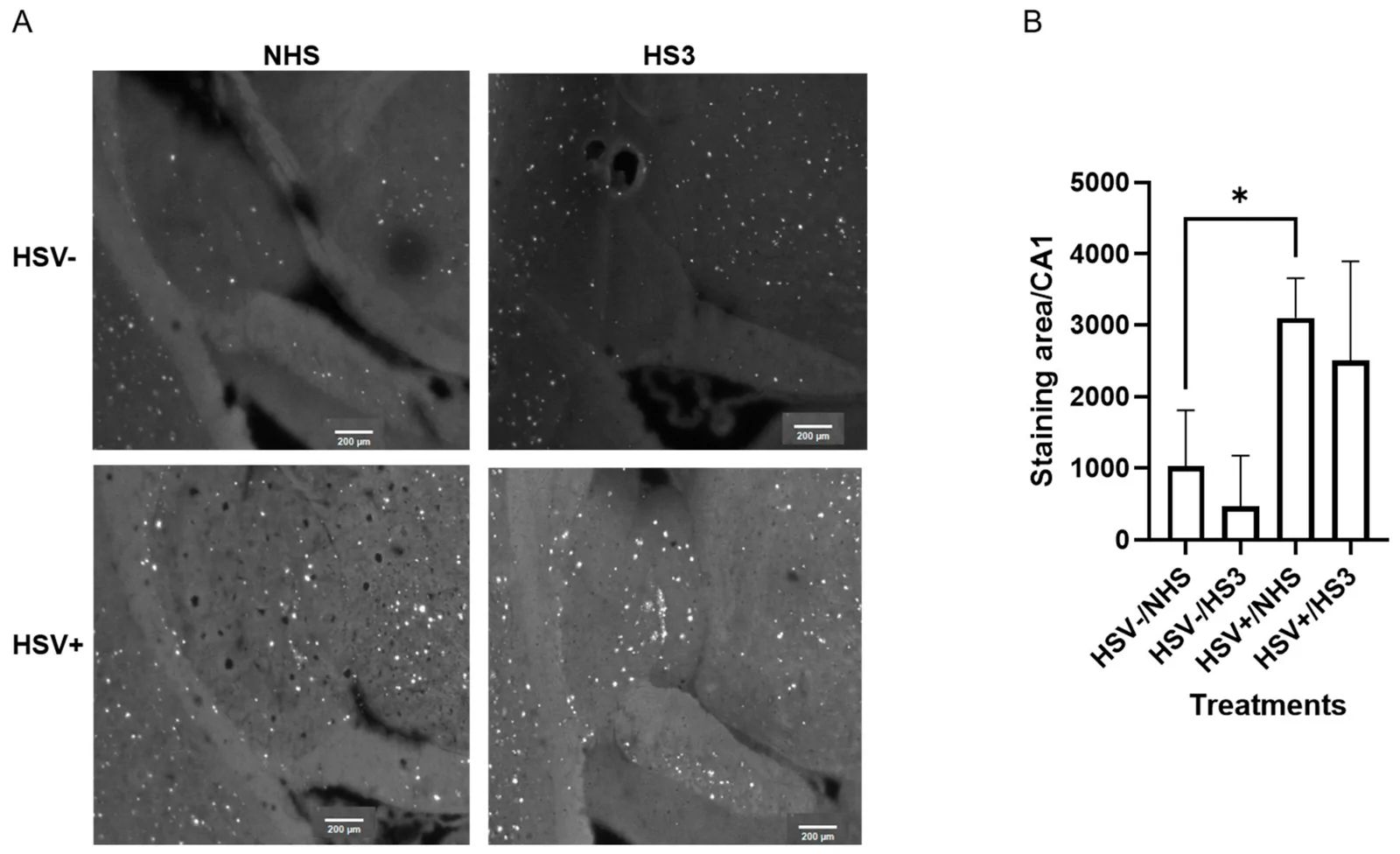
Amyloid plaque staining of the hippocampus from infected (HSV+) and uninfected (HSV-) Het mice. (**A**) Representative images of amyloid plaque staining obtained with a fluorescence microscope under UV light. (**B**) Amyloid plaque staining areas in the CA1 region were measured by ImageJ software. The brain tissue was sectioned at 90 dpi and stained with Amylo-Glo RTD Amyloid Plaque Stain. The image was taken at 25X magnification. The non-stressed HSV-1-infected mice (HSV+/NHS) showed more amyloid staining than HSV-/NHS mice (*p* = 0.02). Means ± SEM. ANOVA and Fisher’s LSD post hoc tests. HS3: HSV-1-infected and uninfected Het mice were stressed at 30, 45, and 60 dpi. N = 3. *: *p* < 0.01.

**Figure 7:**
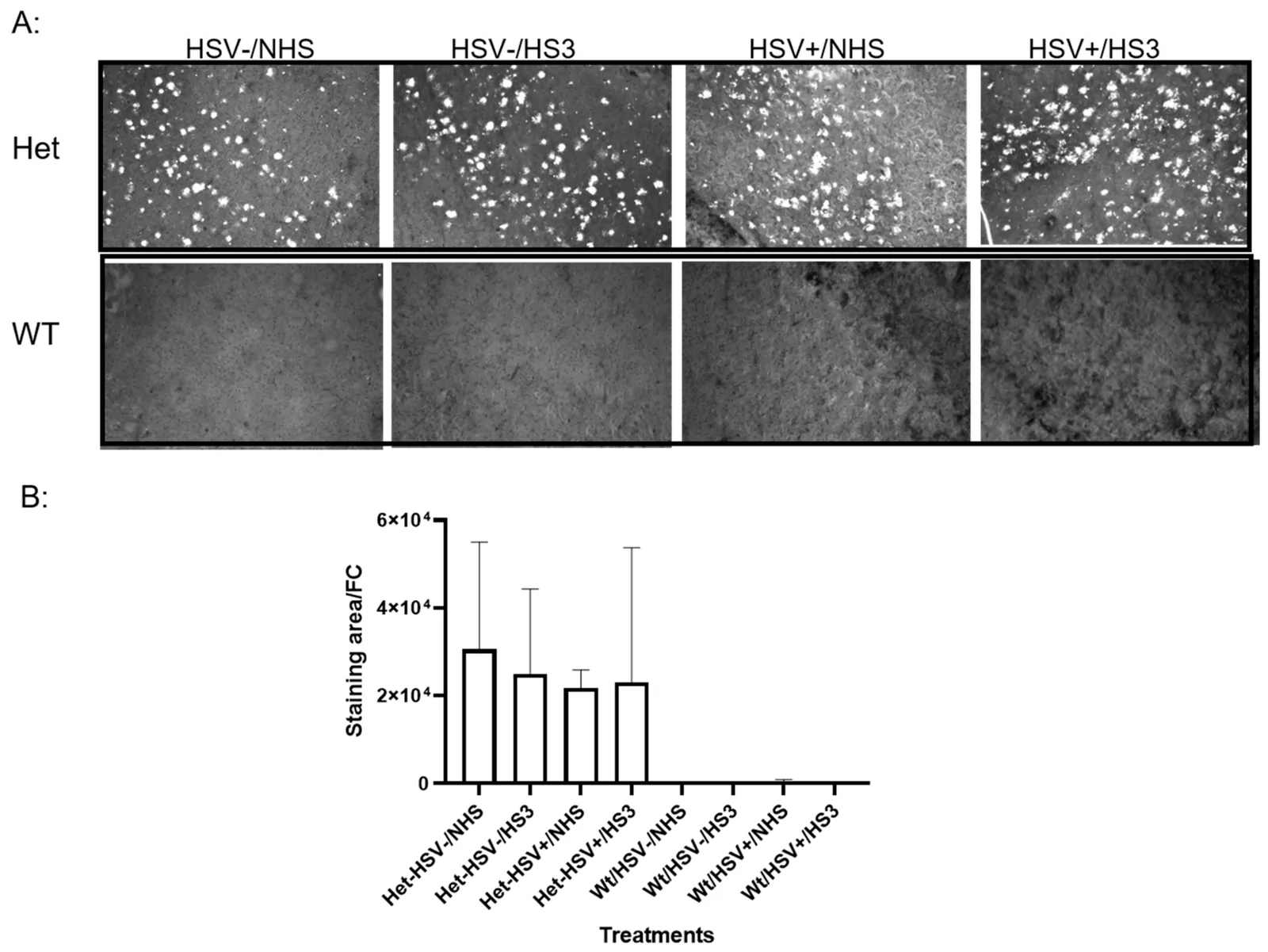
Amyloid plaque staining in the frontal cortex (FC) from infected (HSV+) and uninfected (HSV-) Het and WT mice. (**A**) Representative images of amyloid plaque staining obtained with a fluorescence microscope under UV light. The image was taken at 100X magnification. (**B**) Amyloid plaque staining area in the FC region measured by ImageJ software. The brain tissue was sectioned at 90 dpi and stained with Amylo-Glo RTD Amyloid Plaque Stain. HS3: HSV-1-infected and uninfected Het mice were stressed at 30, 45, and 60 dpi. There were no differences in plaque area in the frontal cortex between treatments in the Het mice. WT mice showed no amyloid plaques in the frontal cortex. Means ± SEM. ANOVA and Fisher’s LSD post hoc tests. N = 3.

To determine whether the differences seen between HSV+ and HSV- Het mice in spatial learning were related to amyloid plaque density in the mouse brain, brain tissues from the three-heat-stress study were harvested 15 days post-behavior tests and sectioned via a Leitz cryostat. Amyloid plaques in three mice from each treatment group were examined by Amylo-Glo staining. The positive staining appeared bright blue under UV light and white in the captured images (Figure 6A and Figure 7A). ImageJ software Version 1.54t 16 May 2026 was used to quantify staining area in the frontal cortex and the Cornu Ammonis 1 (CA1) region of the hippocampus. Three sections from rostral, middle and caudal areas of the brain regions from each mouse were stained, quantitated and averaged as individual staining. Three mice per group were analyzed in each of those regions. The average of the staining per group was compared and analyzed by multiple unpaired *t*-tests in GraphPad Prism 11.0.1. Significantly more amyloid plaque staining in the CA1 region, a critical subfield of the hippocampus involved in spatial memory, autobiographical memory, and memory consolidation, was seen in Het/HSV+/NHS mice than in Het/HSV-/NHS mice (*p* = 0.02; Figure 6B). No significant difference was seen between groups of HSV+ Het mice stressed three times (HSV+/HS3) and HSV+ Het mice without stress (HSV+/NHS). There were no significant differences in amyloid plaque staining in the frontal cortex (FC) region, the brain’s command center for cognitive flexibility, between HSV+ Het mice and HSV- Het mice regardless of stress (Figure 7). No amyloid plaque staining was observed in the brain of WT mice, regardless of infection or stress (Figure 7A).

### 3.5. There Were Increased Inflammatory Cytokines and Reactive Astrocytes in HSV-1 Infected Mice

**Figure 8:**
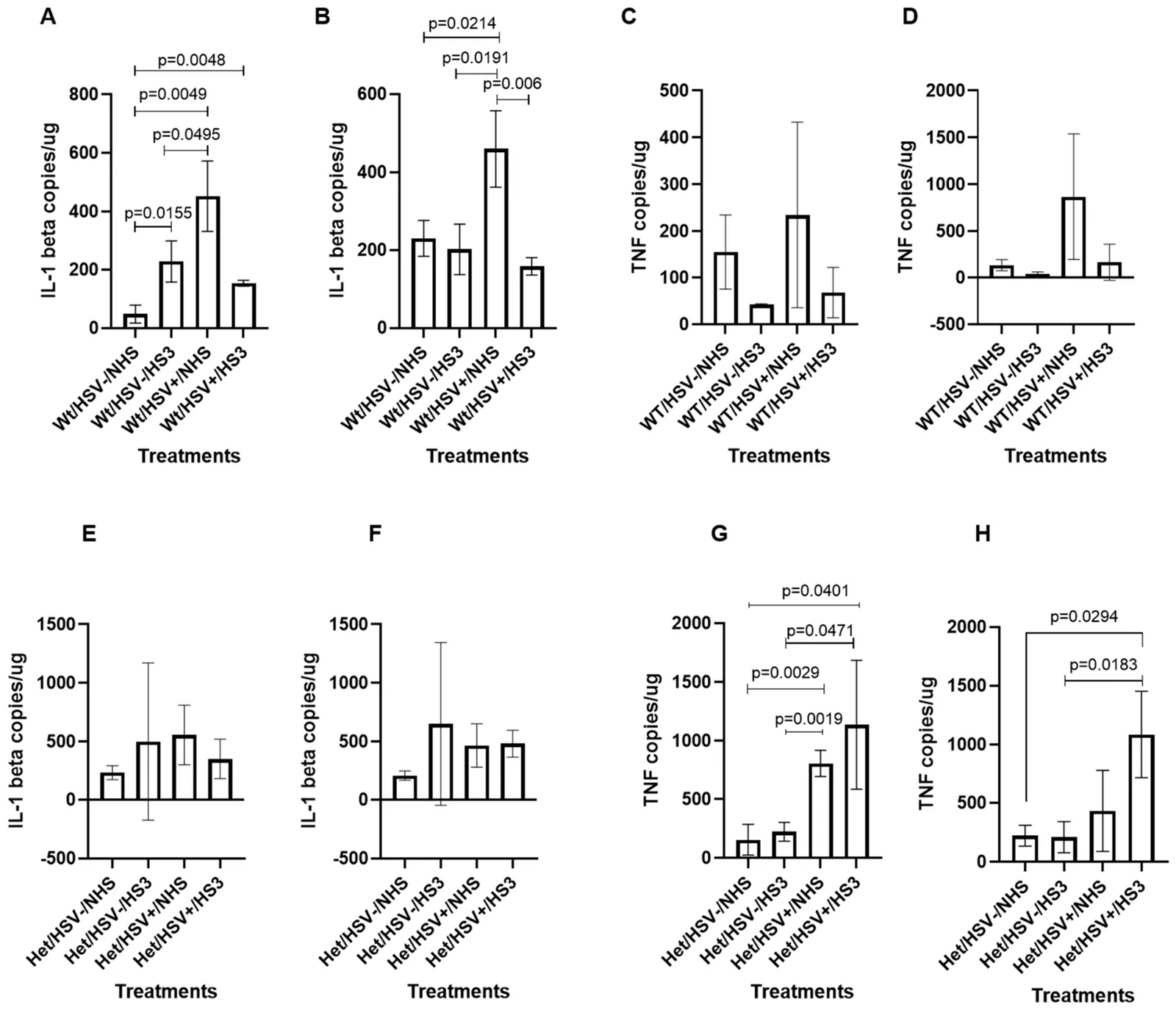
Real-time PCR of IL-1β and TNFα expression in WT (**A**–**D**) and Het (**E**–**H**) hippocampus (**A**,**C**,**E**,**G**) and frontal cortex (**B**,**D**,**F**,**H**) from different treatment groups. The expression of IL-1β and TNFα was measured by SYBR real-time PCR with cDNA generated from total RNA isolated from hippocampus or frontal cortex of Het and WT mice. cDNA was synthesized with 100 ng of total RNA of each tissue from different treatment groups. Transcript copy numbers were estimated by IL-1β and TNFα real-time PCR, respectively, as described in the Materials and Methods Section. Statistical analysis was performed using an unpaired *t*-test. N = 3.

The Amylo-Glo staining demonstrated that there was more beta amyloid in the CA1 region of the hippocampus in Het/HSV+ mice than in the Het/HSV- mice at 90 dpi, which may explain why Het/HSV+ mice had poorer learning performance than the Het/HSV- mice. However, no beta-amyloid staining was observed in WT/HSV+ mice despite also performing worse in the long-term learning trials. A chronic inflammatory response has been reported to be associated with AD-like disease [29]. The innate cGAS-STING inflammatory pathway was found to be activated in an AD-like disease in HSV-1-infected mice [29], suggesting that proinflammatory cytokine genes are activated. To determine whether inflammatory gene transcription was increased in brain tissues harvested two weeks after behavioral testing (at 90 dpi; Figure 5A), transcription of IL-1β and TNF-α in different parts of the brain was examined by reverse-transcription (RT) real-time PCR with primers specific to mouse IL-1β and TNFα. The relative copy number was estimated by a standard curve made with a control plasmid containing the IL-1β and TNFα PCR sequences (Figure S1). In WT mice, HSV+/NHS mice had significantly higher IL-1β gene expression in both the hippocampus (*p* = 0.0049) (Figure 8A) and frontal cortex (*p* = 0.0214) (Figure 8B) than that in uninfected mice. However, no significant difference was seen in TNF-α expression in WT mice regardless of infection or stress in either hippocampus (Figure 8C) or frontal cortex (Figure 8D). In the Het mice, no difference in IL-1β expression was observed between infected and uninfected mice, regardless of stress in the hippocampus (Figure 8E) or the frontal cortex (Figure 8F). However, HSV+/HS3 and HSV+/NHS mice had higher TNF-α expression in the hippocampus than HSV-/NHS and HSV-/HS3 mice (*p* = 0.0401 between HSV+/HS3 and HSV-/NHS, *p* = 0.0471 between HSV+/HS3 and HSV-/HS3, *p* = 0.0029 between HSV+/NHS and HSV-/NHS, *p* = 0.0019 between HSV=/NHS and HSV-/HS3) (Figure 8G), while only HSV+/HS3 mice had significantly higher TNF-α gene expression than HSV-/NHS and HSV-/HS3 mice in the frontal cortex (*p* = 0.0294 and *p* = 0.0183, respectively) (Figure 8H).

**Figure 9:**
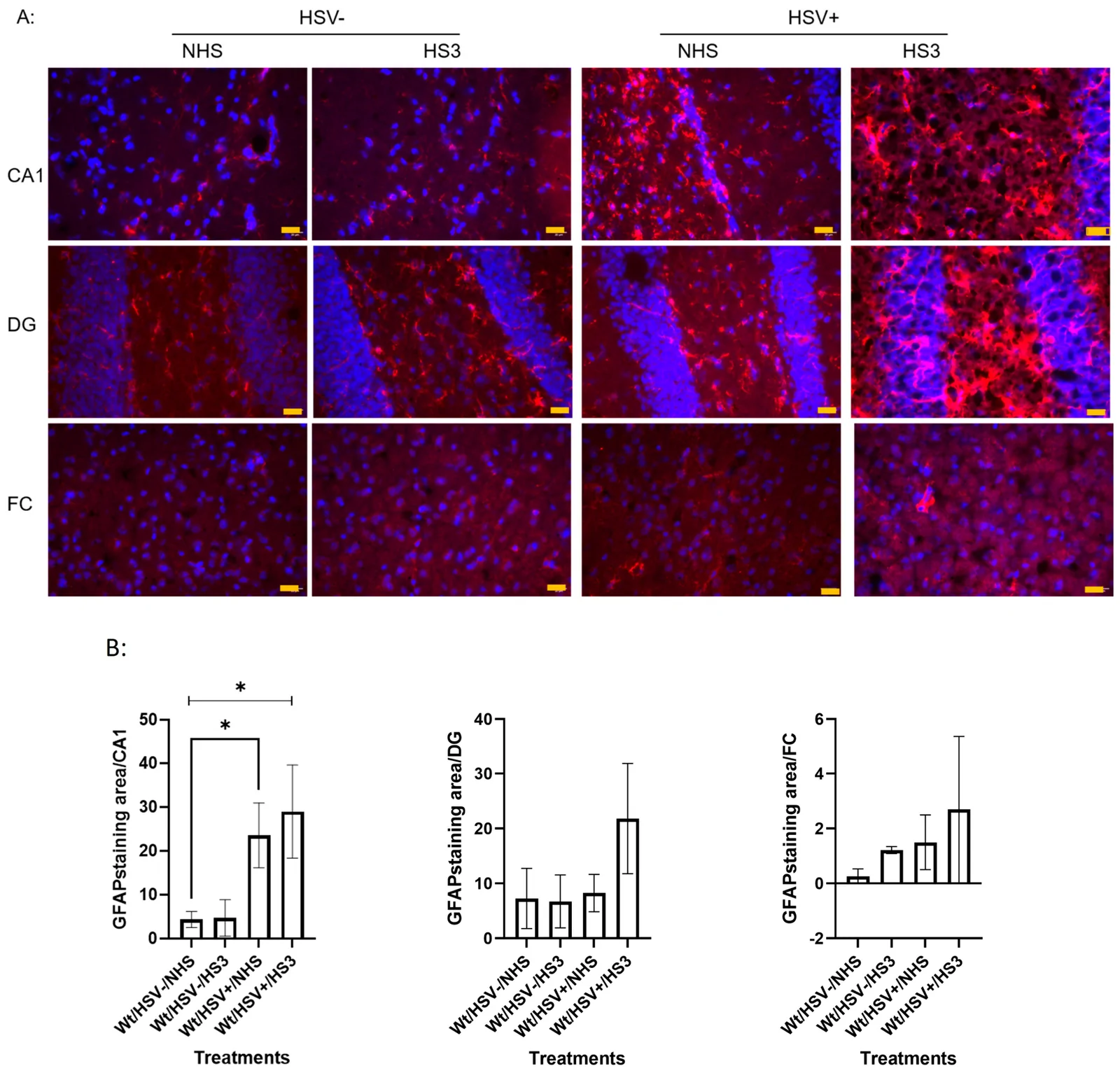

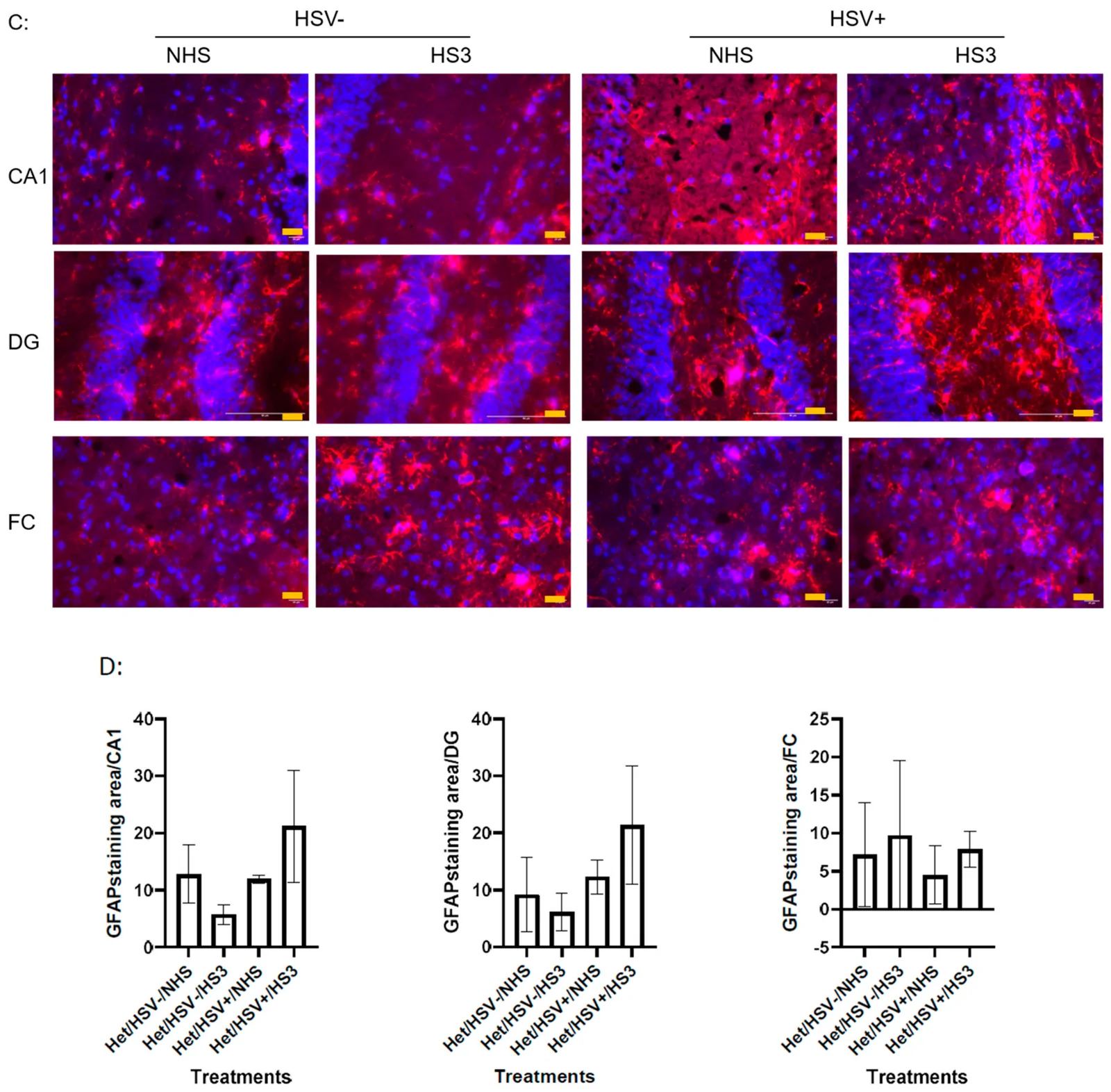
Indirect immunofluorescence analyses of brain sections from WT (**A**,**B**) and Het (**C**,**D**) mice. (**A**,**C**) GFAP antibody staining (red) of brain sections from hippocampus (CA1 and DG) and frontal cortex (FC) from WT (**A**) and Het (**C**) mice. Brain sections were fixed, permeabilized in acetone first, then stained with rabbit polyclonal GFAP antibody first, then goat anti-rabbit secondary antibody. Cell nuclei were visualized with DAPI (blue). CA1, DG, and FC correspond to the Cornu Ammonis 1 region of the hippocampus, the dentate gyrus, and the frontal cortex, respectively. The image was taken at 400X magnification. (**B**,**D**) Quantification of GFAP staining area in CA1 region from WT (**A**) and Het (**C**) mice measured by ImageJ software. HS3: HSV-1-infected and uninfected Het mice were stressed at 30, 45, and 60 dpi. Statistical analysis was performed by using multiple unpaired *t*-tests, *: *p* < 0.01, N = 3. Scalebar = 20 µm.

Inflammatory responses to brain injury and neurodegeneration are often accompanied by reactive astrocytes (gliosis), which have increased GFAP expression. To determine if there were differences in reactive astrocytes in the brain, brain sections from all treatment groups were stained with GFAP antibodies. Although few reactive astrocytes were observed in the FC regions in the HSV-1-infected WT mice, more GFAP staining was observed in the CA1 and dentate gyrus (DG) regions in the infected WT mice (Figure 9A). The GFAP staining in the CA1 region in the WT/HSV+/NHS and WT/HSV+/HS3 mice was significantly higher than in the WT/HSV-/NHS mice (Figure 9B). On the other hand, increased GFAP staining area was seen in FC, CA, and DG regions in both infected and uninfected Het mice (Figure 9C). There was no significant difference in GFAP staining between Het/HSV+ and Het/HSV- mice in the CA1 region, FC and DG regions (Figure 9D).

### 3.6. HSV-1 Genome Was Detected in Many Regions of the Infected Mice at 90 Dpi

**Figure 10:**
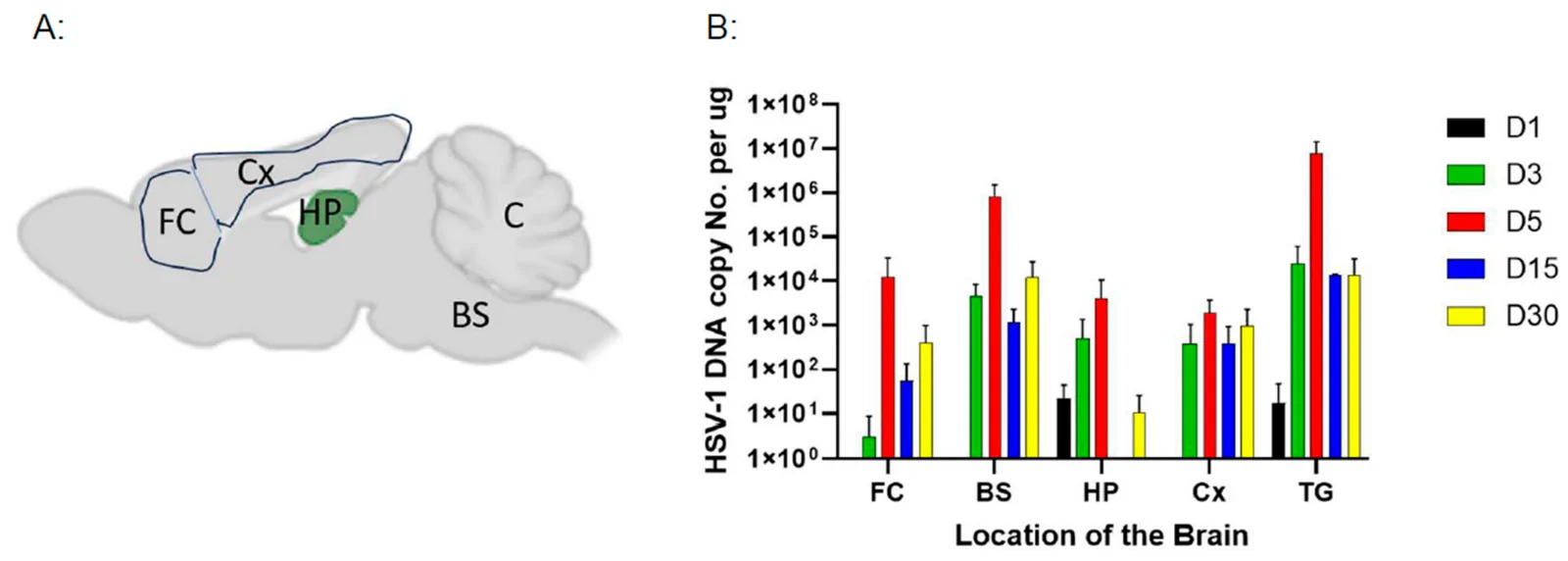
Sagittal brain section and real-time PCR of HSV-1. (**A**) Sagittal brain section of the mouse brain (made by BioRender) showing regions sampled. Labeled brain regions (frontal cortex (FC), caudal cortex (Cx), hippocampus (HP), cerebellum (C), and brainstem (BS)) were collected for HSV-1 DNA or RNA analysis. (**B**) HSV-1 DNA copies per ug tissue estimated by real-time PCR with primers specific for HSV-1 LAT gene. TG: Trigeminal ganglion. D1, D3, D5, D15, and D30 correspond to 1, 3, 5, 15, and 30 days post-infection, respectively. N = 3.

**Figure 11:**
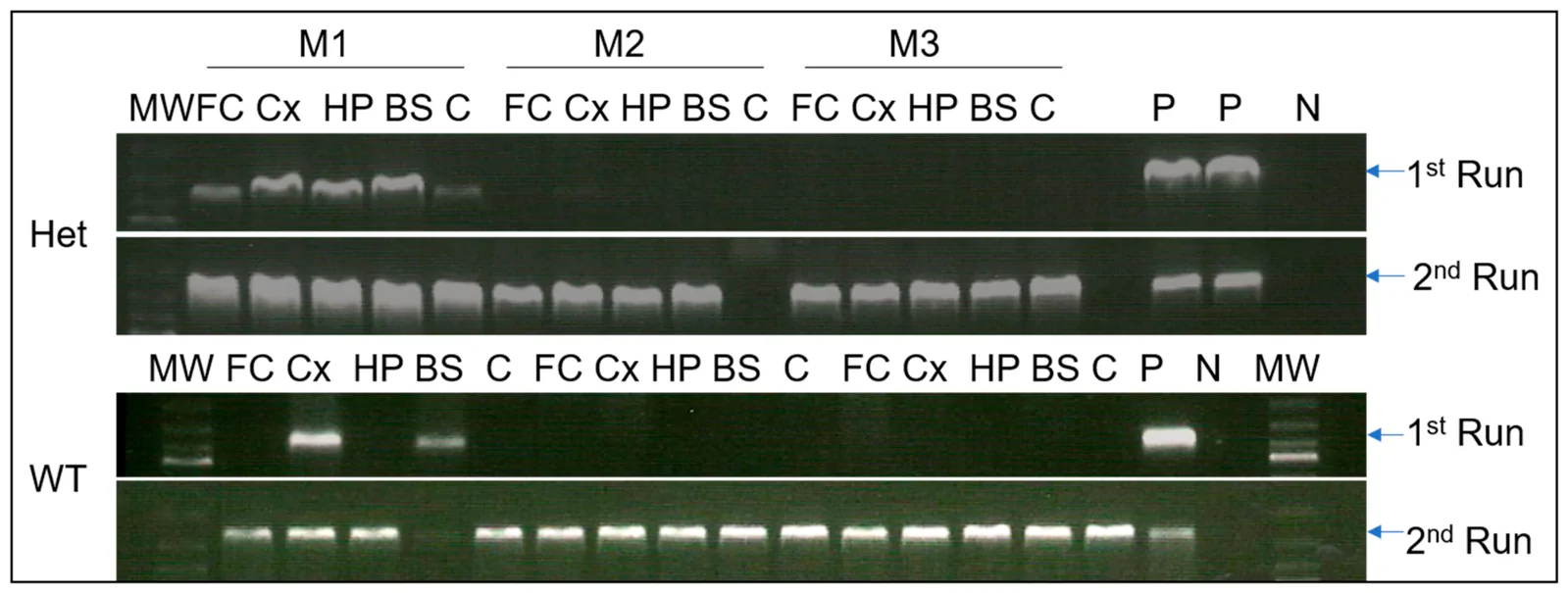
Detection of HSV-1 DNA in the brain tissues by nested PCR with primers specific to UL36. The 1st-run PCR product was amplified from total DNA extracted from brain tissues harvested at 90 dpi from Het (top two images) and WT (bottom two images) mice. The 2nd-run PCR product was amplified from the 1st-run PCR product for each sample. Lane labels: MW, 1 kb plus ladder; FC, frontal cortex; Cx, caudal cortex; HP, hippocampus; BS, brainstem; C, cerebellum; P, HSV-1 genome; and N, uninfected tissue DNA. M1-3 represent HSV-1 DNA detection in different HSV-1+/NHS mice. N = 3.

To understand why inflammatory gene expression was present in the mouse brain at 90 dpi, we hypothesized that HSV-1 was not completely cleared following an acute infection, and residual viral components may trigger chronic inflammation in the brain. To determine whether HSV-1 DNA persists in the brain after exposure, HSV-1 DNA was examined directly in different parts of the brain (Figure 10A) harvested during the acute phase and latent phase. Total DNA was isolated from frontal cortex (FC), caudal cortex (Cx), hippocampus (HP), brainstems (BS) and cerebellum (C) of three WT mice. HSV-1 DNA was quantitated by real-time PCR specific for the LAT gene at 1, 3, 5, 15 and 30 days post-infection (dpi). As shown in Figure 10B, viral DNA was barely detectable at 1 dpi; by day 3, it was detectable in most of the brain regions examined. Viral DNA peaked around day 5 post-infection and remained detectable through 30 dpi in the tested tissues. To determine whether HSV-1 persists in the brain at 90 dpi, HSV-1 DNA was first examined in total DNA isolated from HSV-infected and unstressed brain tissues by nested PCR using primers specific to UL36. Although there were few or no detections in the first-run PCR, HSV-1 DNA was amplified in the 2nd-run PCR reaction using the first-run PCR products in many brain sections, regardless of genotype (Figure 11). To determine if the HSV-1 genome increased in mice that experienced three heat stresses, HSV-1 DNA was monitored by real-time PCR specific to LAT or gD. As shown in Figure S5, no significant differences were observed in HSV-1-infected mice regardless of stress in either Het or WT mice.

### 3.7. HSV-1 Became Latent in Many Regions of the Brain in the Infected Mice

**Figure 12:**
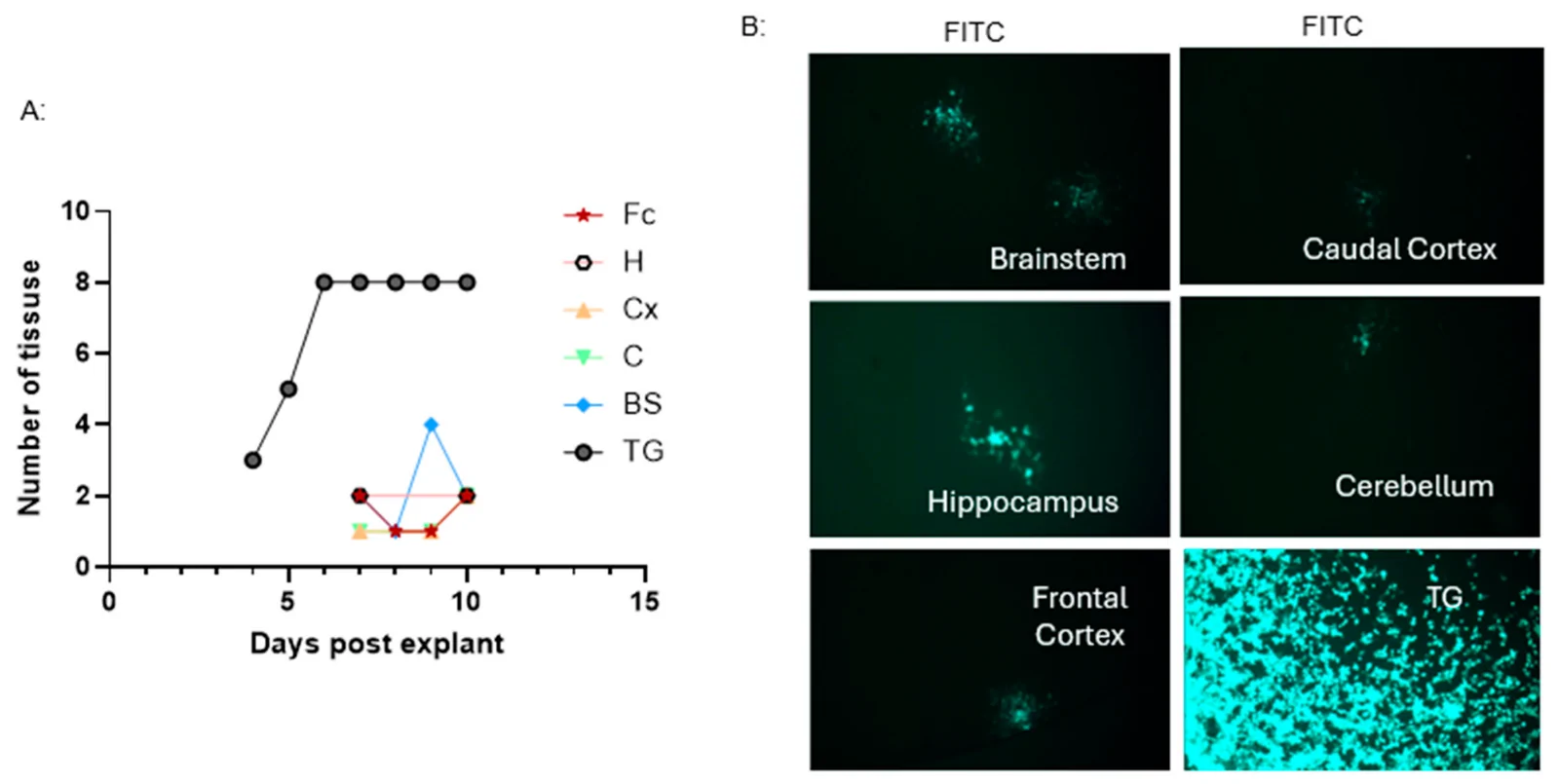
HSV-1 reactivation via mouse brain tissue explant culture. (**A**): Number of tissues with HSV-1 reactivation in tissue explant cultures. Brain tissues (frontal cortex (Fc), caudal cortex (Cx), hippocampus (H), cerebellum (C), brainstem (BS), and trigeminal ganglia (TG)) were harvested at 33 days post-infection and cultured in a 12-well plate in 1.5 mL DMEM with 10% FBS. (**B**): GFP-positive cells infected with tissue explant medium on day 7 post-explant. Images were taken at 24 h post-infection with a 10x FITC fluorescent microscope. N = 8.

The results above demonstrated that HSV-1 DNA persisted in different regions of the mouse brain after recovery from HSV-1 infection. It is possible that those viral genomes could become latent in brain neurons. To determine whether HSV-1 becomes latent in the brain, additional WT mice were infected with HSV-1 as described in the Materials and Methods. Frontal cortex (Fc), caudal cortex (Cx), hippocampus (H), cerebellum (C), and brainstem (BS) from eight latently infected mice were explanted at 33 days post-infection and cultured in 1.5 mL DMEM in a 12-well plate as we described previously [33]. By day 4 post-tissue explant, three out of eight TG explants started producing infectious virions; by day 6 post-tissue explant, CPE was seen in RS cells infected with TG explant culture media from eight out of eight mice (Figure 12A, Table 2). The CPE was verified by GFP expression (green) in the infected RS cells (Figure 12B). By day 7 post-tissue explant, HSV-1 reactivation was also observed in Fc, H, Cx, and C explant culture media (Figure 12A, Table 2). Between day 7 and 10 post-tissue explant, HSV-1 reactivation was observed in six out of eight brainstems, six out of eight frontal cortexes, five out of eight caudal cortex explants, four out of eight hippocampal and cerebellar explants (Table 2). The amount of virus reactivated from the brain explant was relatively lower compared to the amount of virus from the TG explant. A significantly higher level of GFP expression was observed in RS cells infected with TG explant media at 24 hpi. In contrast, relatively few infection foci were observed in cells infected with brain tissue explant media (Figure 12B).

## 4. Discussion

Alzheimer’s disease (AD) is a disease that involves several different etiopathogenic mechanisms [41,42,43]. In this study, we tested the infectious theory of AD in an HSV-1 mouse model using Heterozygous 5XFAD mice (Het) carrying 5 AD-linked mutations on a C57BL/6 background and wild-type littermates (WT). Het mice are commonly used in Alzheimer’s disease model, and are characterized by rapid, age-related amyloid plaque accumulation, robust neuroinflammation, and significant memory deficits starting at 4–6 months [32]. These Het mice, like WT mice, were susceptible to HSV-1 infection. Both Het and WT mice developed ocular disease following HSV-1 inoculation during the acute infection phase. Only 5–10% of them died from the acute HSV-1 infection when they were infected with a high infectious dose of around 2X10^5^ PFU/eye; the majority survived the acute infection. HSV-1 can become latent in the TG of the Het mice, as in WT mice. Lytic viral gene transcription in the latently infected brain was observed following heat stress within 3 days post-heat stress in both Het and WT mice (Figure 2). Therefore, the effect of heat stress on HSV-1 persistency and AD-related disease was evaluated in Het mice in this study.

It is not well understood why HSV-1 is associated with AD. We hypothesized that HSV-1 components could persist in the infected brain and that multiple heat stresses would cause greater HSV-1 persistence, cognitive decline, and AD-like pathology. We expected that infected mice that experienced heat stress during latency would have worse cognitive function. Infection alone interfered with learning in the water maze, regardless of genotype or stress, suggesting that negative effects of the initial infection can be long-term. Stress led to poorer memory in the probe trials 30 days post-infection across genotypes, but Het mice alone were negatively affected by the second stress. The poorer performance following heat stress in behavioral trials observed in this initial study could have been due to lytic HSV-1 replication, since the behavioral testing was performed 3 days after heat stress (Figure 3 and Figure 4), which can cause HSV-1 reactivation. This suggests HSV-1 lytic replication could stimulate cognitive declines as reported previously [24].

To rule out the possibility of lytic HSV-1 replication from heat stress during the behavior test, additional groups of mice were infected and stressed zero to three times at 30, 45, and 60 dpi, as indicated in Figure 5A, and cognitive testing was performed 15 days after the final heat stress to ensure that the mice exposed to multiple stresses and non-stressed mice were all evaluated during the same latency period. These mice were about 4.5 months old when they were tested in the water maze. Interestingly, HSV+ mice performed worse than HSV- mice in learning trials when the behavior tests were run at 75 dpi (Figure 5). The amyloid plaque staining showed more amyloid plaque staining in the CA1 region of the hippocampus in Het/HSV+/NHS mice (Figure 6). These results suggest that HSV-1 latency alone may enhance AD-like pathology and negatively affect cognitive function.

We hypothesized that latently infected mice that experienced more heat stresses would have more amyloid plaques, more viral products in the brain and reduced cognitive function. However, amyloid staining in Het mice did not support our hypothesis. At 90 dpi, Het/HSV+/NHS mice had significantly more amyloid plaques than Het/HSV-/NHS mice in the CA1 region of the hippocampus (Figure 6). However, Het/HSV+/HS3 had relatively fewer amyloid plaques in the CA1 region compared to those in Het/HSV+/NHS mice. A similar trend was observed between Het/HSV-/NHS and Het/HSV-/HS3 mice, although the difference was not significant. It is known that heat stress can lead to a wide range of host stress responses and production of different heat shock proteins [44]. Heat shock proteins (Hsps) induced by heat stress can prevent protein misfolding and are capable of clearing aggregated or damaged proteins from cells [45,46]. It has been reported that induction of Hsps could reduce tau pathology and amyloid plaque formation in transgenic AD mice [47]. In a 2016 publication, Kasza et al. demonstrate that dihydropyridine derivatives (such as LA1011) as Hsp co-inducers can provide neuroprotective and disease-modifying treatment for AD [48]. Another study reported in 2017 found that recombinant heat-shock protein HSP70 can reduce the elevated intracellular proteasome activity induced by isomerized amyloid-beta (Aβ42) in human SK-N-SH neuroblastoma cells [47]. Therefore, heat stress used in our study may have triggered Hsps production, which may reduce amyloid peptide production in stressed Het mice. This may explain why stressed and unstressed Het mice had no significant difference in amyloid plaque staining in the infected mice during latency (Figure 6 and Figure 7).

No amyloid plaques were observed in WT mice at 90 dpi, regardless of infection or heat stress. This raised the question of why infected WT mice also show decreased spatial learning (Figure 5B). A 2024 study found that chronic inflammatory responses to HSV-1 infection were associated with increased tau phosphorylation and the presence of ICP27 [29]. To determine whether chronic inflammation plays a role in memory decline in WT mice, tissues harvested at 90 dpi were examined for IL-1β and TNF-α gene transcription. Interestingly, increased IL-1β was observed in both frontal cortex tissue and hippocampus tissue from the infected WT mice; however, TNFα gene transcription was not significantly different between HSV+ and HSV- WT mice (Figure 8). IL-1β is a potent, pro-inflammatory cytokine and a key mediator of the innate immune response, acting as a crucial “alarm” signal during infection, injury, and stress [49,50]. The difference in IL-1β expression suggests an inflammatory response is taking place in WT/HSV+ mice. The increased inflammation in the hippocampus may contribute to the spatial learning deficits in WT mice (Figure 5B).

The difference in IL-1β expression is less significant between infected and uninfected Het mice. Het mice are reported to have lower IL-1β expression around 4 months of age in male mice than in female mice, which may explain the lack of a significant difference, since our investigation used mixed-sex mice [51]. In contrast, TNF-α mRNA transcription differed significantly in the hippocampus between HSV+ and HSV- Het mice. In Het mice, TNF-α transcription is known to be higher at 5 months of age, regardless of sex [44]. HSV-1 infection may further increase TNF-α transcription in Het mice. TNF-α is a master pro-inflammatory cytokine that drives low-grade chronic inflammation and facilitates persistent, low-level systemic inflammation [52,53,54]. A combination of low-grade inflammation and amyloid plaque accumulation may drive the decline in spatial learning in Het/HSV+ mice.

Because the IL-1β and TNFα genes were upregulated in HSV+ mice, we expected more reactive astrocytes in the HSV+ mouse brain. Reactive astrocytes are CNS glial cells characterized by hypertrophy and increased GFAP expression in response to injury, disease, or infection. They are key players in neuroinflammation and stroke, often releasing toxic factors or, conversely, aiding in repair [55]. Reactive astrocyte responses are driven by signals such as the cytokines IL-1β and TNFα and can be sustained for long periods [56]. Using GFAP antibody staining, increased GFAP staining was observed in the frontal cortex and hippocampus of Het mice, regardless of infection (Figure 9C,D). Although very little GFAP staining was observed in the frontal cortex of WT mice, there was significantly more GFAP staining in the WT/HSV+ mice than in the WT/HSV- mice in the CA1 region (Figure 9B). These results suggest that greater hippocampal neuroinflammation in WT/HSV+ mice may contribute to spatial learning deficits.

To understand what drives the inflammatory response in infected mice, we hypothesized that HSV-1 components, such as the viral genome or proteins, were not fully cleared from the brain and latently infected mice experiencing more heat stress would have more viral DNA or viral protein persistence in the brain. To determine whether viral proteins persisted, we stained brain sections harvested at 90 dpi with antibodies specific to HSV-1 ICP27 and GFAP. We did not consistently detect ICP27 in infected Het and WT mice (Figures S6 and S7). Only 1 out of 3 positive ICP27 stainings was observed in the DG and CA1 regions in Het/HSV+ mice (Figure S6, white arrow), and 1 out of 3 positive stainings in the CA1 region in WT/HSV+ mice (Figure S7, green arrow). Additional ICP27 co-staining with FJC staining specific for degenerated neurons revealed relatively low ICP27 staining in HSV+ mice regardless of stress or genotype. However, strong ICP27 staining co-localized with FJC staining in 1 of 3 WT/HSV+/NHS mice (Figure S8). These results suggest that viral protein did persist in the latently infected brain, but heat stress did not increase viral protein accumulation in our current study.

We examined viral DNA persistence in different brain regions using nested PCR and real-time PCR. In agreement with our hypothesis, viral DNA was detectable in the frontal cortex, caudal cortex, hippocampus, and cerebellum, as well as in the TG and the brainstem, at 90 dpi in both stressed and unstressed mice (Figure 11 and Figure S5). Like the viral protein staining observation, viral genome copy number in mice that experienced heat stress was not different from infected mice without heat stress. HSV-1 reactivation may vary by host and by heat stress, and not all animals will experience the same level of reactivation; therefore, it is not surprising to find no correlation between stressed and unstressed animals in viral genome copy numbers (Figure S5).

It is unknown whether HSV-1 can become latent in neurons of the cerebrum and cerebellum and reactivate from latency. Data on HSV-1 reactivation from latency in the cerebrum have been contradictory to date [57,58]. Our in vitro tissue explant culture showed that infectious viruses could be reactivated from multiple regions of the brain (Figure 12 and Table 2). However, HSV-1 reactivation frequency and magnitude in the brain and brainstem were relatively lower than those in the TG (Figure 12A and Table 2). It is common knowledge that no viral protein is expressed during HSV-1 latency in the TG. Our study demonstrated that both the viral genome and the viral protein, ICP27, were present in the brain 90 dpi. ICP27 was also reported to be present in severe AD [29]. It is possible the HSV-1 latency in the brain is an “aberrant latency” which is different from the HSV-1 latency in the TG. This aberrant latency may play a role in AD pathology and cognitive decline.

Limitations of the study: It is known HSV-1 latency can be established in the TG in recovered animals. However, only 1–10% of neurons are latently infected in the TG [59,60]. HSV-1 reactivation from latency also varies between hosts and neurons. HSV-1 reactivation may differ between TG neurons and brain neurons. Therefore, it is likely that not all mice that experienced heat stress will undergo reactivation. It is also possible that the three heat stresses did not trigger significant HSV-1 reactivation; therefore, we observed no significant difference between stressed and unstressed mice after recovery from stress. It is also possible that our hypothesis that multiple heat stresses will cause more HSV-1 persistence is untrue. Regarding ICP27 staining, we examined only three sections from each brain per animal; therefore, we may have missed sections with persistent HSV-1. In addition, the small N for behavioral assays likely limited the ability to detect significant interactions.

## 5. Conclusions

In conclusion, our study found that HSV-1 infection persisted in the brain and may become aberrantly latent in many brain regions, which is different from HSV-1 latency in the TG. This aberrant latent infection may cause increased amyloid plaque production and increased TNF-α expression in Het mice and increased IL-1β expression and more reactive astrocytes in WT mice and may influence learning and memory in both.

## Figures and Tables

**Table 1:** Primers for reverse transcription PCR and 5XFAD screening.

Name	Gene	Sequence (5′-3′)
IL-1βF	IL-1β	GCAGGCAGTATCACTCATTGTGG
IL-1βR		GAGTCACAGAGGATGGGCTCTTC
TNF-F	TNF-α	GGCAGGTCTACTTTGGAGTCATTGC
TNF-R		ACATTCGAGGCTCCAGTGAATTCGG
FAD37598-cF	FAD typing	ACCCCCATGTCAGAGTTCCT
FAD27367-mR		CGGGCCTCTTCGCTATTAC
FAD37599-wtR		TATACAACCTTGGGGGATGG
gD-FOR	gD	GCGGCTCGTGAAGATAAACG
gD-REV		CTCGGTGCTCCAGGATAAACTG
gD-Probe		56-FAM/CTGGACGGA/ZEN/GATTACA/3IABkFQ/
UL36-F351	UL36	ACAGTGTTGGTGGTGTCCTG
UL36-R351		CGCGGATCAGGTCGAAATTC
UL36-F253		GAAGCGACCCCATACCTCG
UL36-R253		TCAGGTCGAAATTCTGGTGGA
LAT-F	LAT	GGGTGGGCTCGTGTTACAG
LAT-R		GGACGGGTAAGTAACAGAGTCTCTA
LAT-Taqman probe		FAM-ACACCAGCCCGTTCTTT-3′

**Table 2:** Number of tissues with infectious virus production during in vitro tissue explant culturing.

Days Post-Explant	Frontal Cortex	Hippocampus	Caudal Cortex	Cerebellum	Brainstem	TG
1						
2						
3						
4						3
5						5
6						8
7	2	2	1	1	1	8
8	1		1		1	8
9	1		1	1	4	8
10	2	2	2	2	1	8
Total tissue postive	6	4	5	4	6	8

## Data Availability

The original contributions presented in this study are included in the article/Supplementary Material. Further inquiries can be directed to the corresponding authors.

## Supplementary Materials

The following supporting information can be downloaded at: https://www.mdpi.com/article/10.3390/cells15201841/s1.

## Abbreviations

AD	Alzheimer’s disease
Het	5 × FAD heterozygotes
HSV-1	Human Herpes Simplex type 1 virus
WT	Wild-type mice
FC	Frontal cortex
CA1	Cornu Ammonis 1 region
GFAP	Glial Fibrillary Acidic protein
ICP27	Immediate-early or α protein 27
Ab	Amyloid beta
TG	Trigeminal ganglia
RS	Rabbit skin cells
GFP	Green Fluorescent Protein
PCR	Polymerase Chain Reaction
NHS	No heat stress
HS	Heat stress
