# Supplementary material for: Differential Genotypic Effects of HSV-1 Infection on Cognitive Function and Pathology in the 5xFAD Mouse Model
Source: Cells. 2026 Oct 10;15(20):1841. doi: 10.3390/cells15201841 (PMC13702985; doi:10.3390/cells15201841)
Supplement: Supplementary file 1 [file cells-15-01841-s001.zip › cells-4481265-supplementary.pdf]

Supplementary Materials:

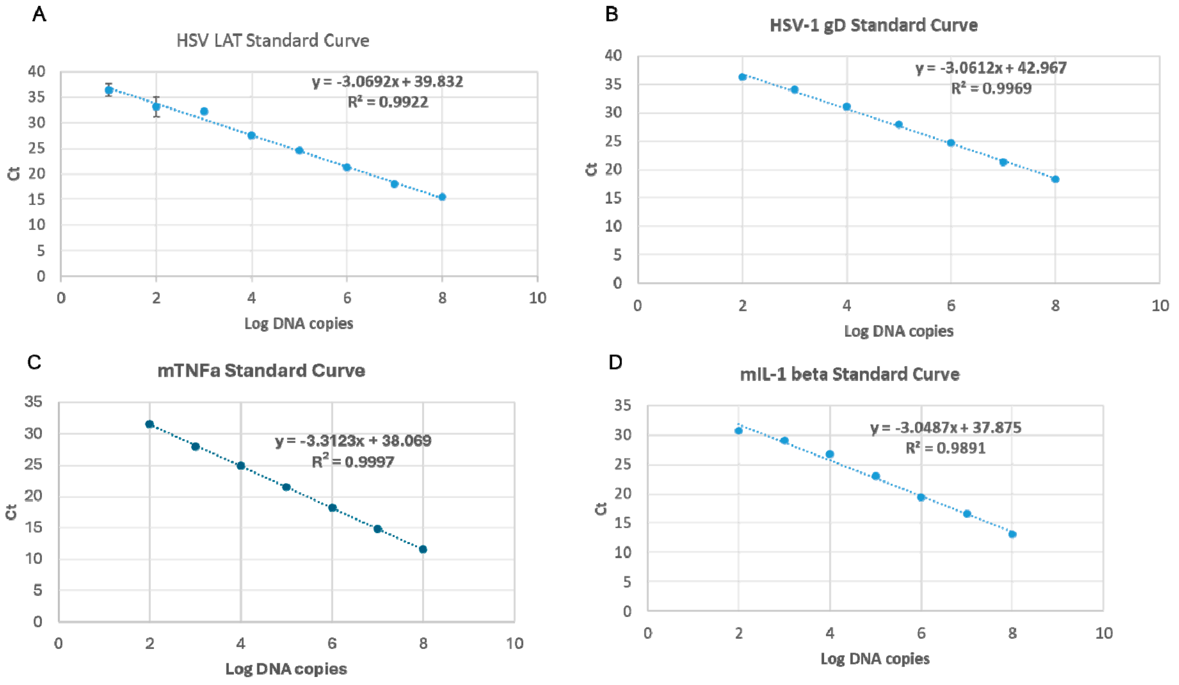

Sup Figure S1. Real-time PCR Standard curve using threshold cycle values to calculate viral genome copies in the real-time TaqMan PCR (A and B) and SYBR PCR (Cand D). The assay detects a range from 10 to 10^8 copies of a plasmid bearing the LAT (A), gD (B), mTNFa (C) and mIL-1 beta (D) target sequences.

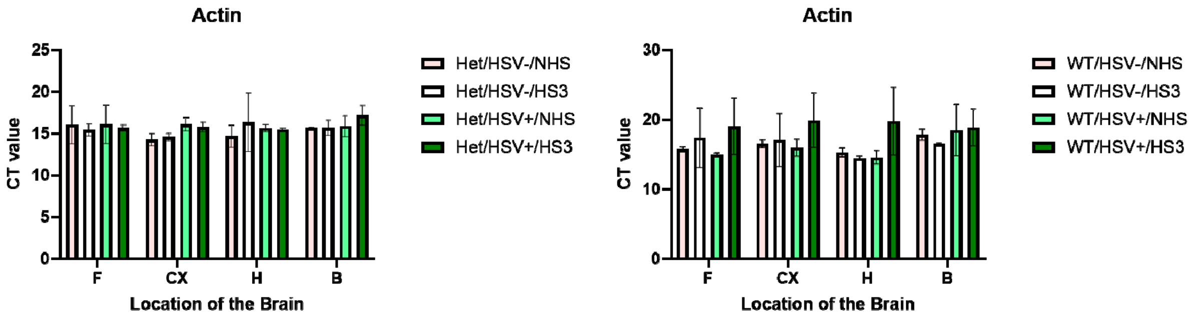

Sup Figure S2. Real-time PCR of beta-actin in brain tissues collected from Het (left) and WT (right) mice. Real-time PCR of beta-actin, specific to the beta-actin gene. C and D: FC, Cx, H, C, and BS correspond to the frontal cortex, caudal cortex, hippocampus, cerebellum, and brainstem, respectively. HSV-: uninfected

mice; HSV+: infected mice; NHS: no heat stress; HS: stress three times at 30, 45, and 60 dpi, respectively. N=3

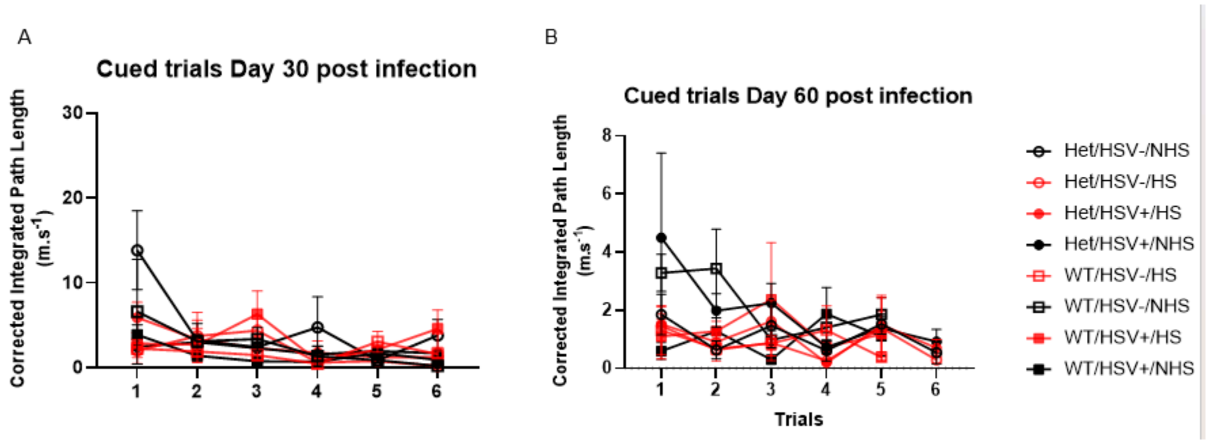

Sup Figure S3. Associative memory cued control trials in both WT and Het mice that experienced heat stress once at 30 dpi (A) and twice at 30 and 60 dpi (B). Mice were stressed and tested using a Morris water maze, starting 3 days post-heat stress at 42°C for 10 min. HSV+: HSV-1 infected mice, HSV-: uninfected mice; HS: stressed twice, NHS: unstressed. Means ± SEM. RANOVA. N = 3-6.

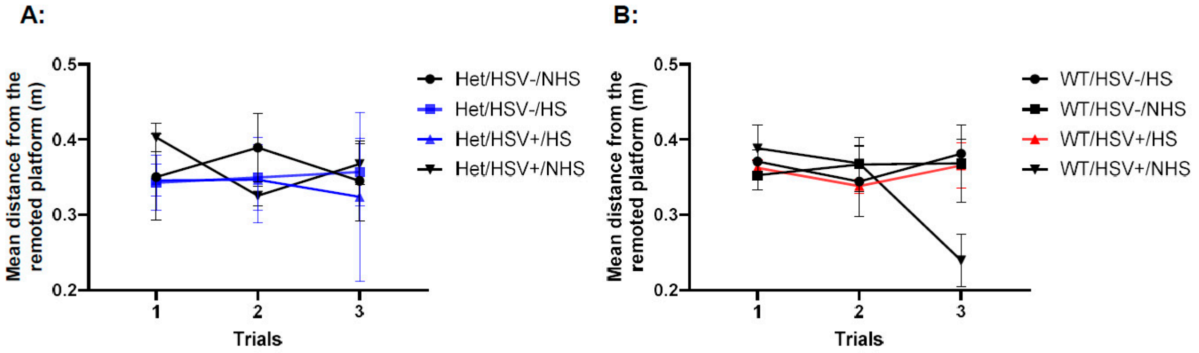

Sup Figure S4. Long-term memory (probe trial) in Het and WT mice. Mice were stressed twice on days 30 and 60 post-infection and tested in a Morris water maze 3 days post-heat stress (42 C; 10 min). HSV+: HSV-1 infected mice, HSV-: uninfected mice; HS: stressed twice, NHS: unstressed. Means ± SEM. RANOVA. N = 3-6.

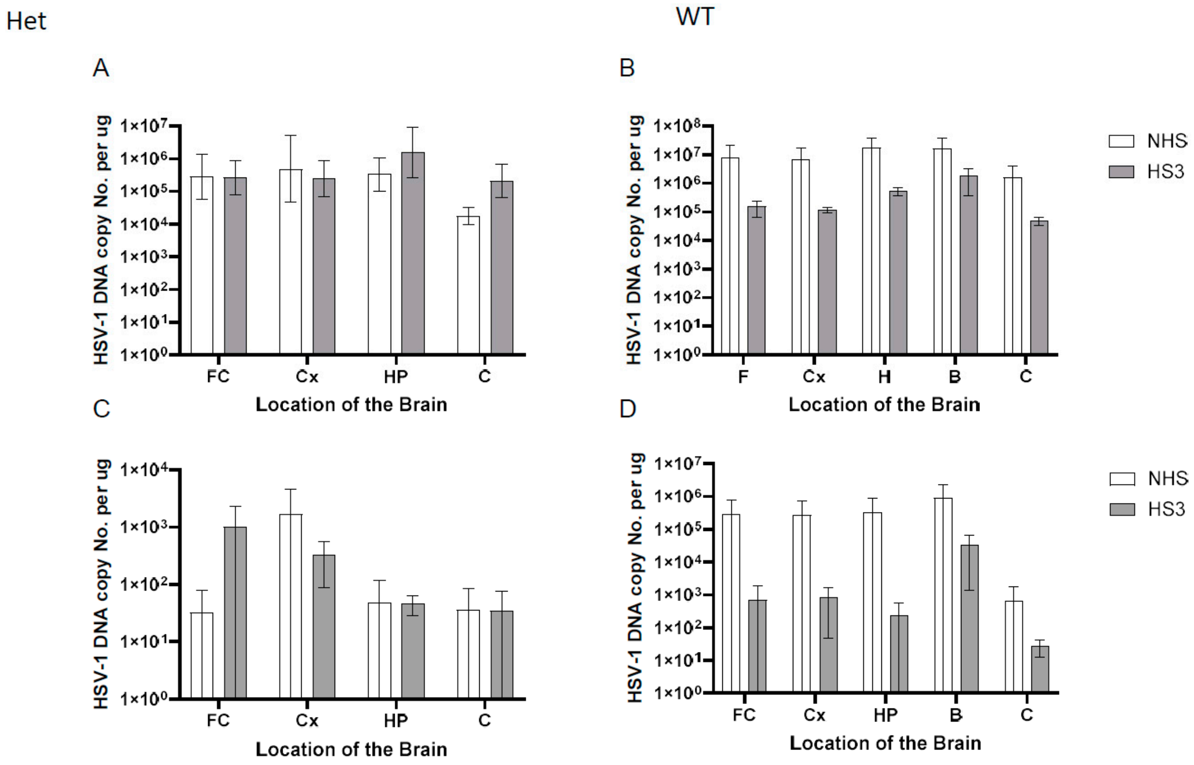

Sup Figure S5. Real-time PCR of HSV-1 in the brain tissues collected from Het (A and C) and WT (B and D) mice. A and B: Real-time PCR of HSV-1 specific to the gD gene. C and D: Real-time PCR specific to the HSV-1 LAT gene. FC, Cx, H, C, and BS correspond to the frontal cortex, caudal cortex, hippocampus, cerebellum, and brainstem, respectively. The open bar represents viral DNA detected in infected mice without stress; the gray bar represents viral DNA detected in infected mice stressed 3 times. N=3

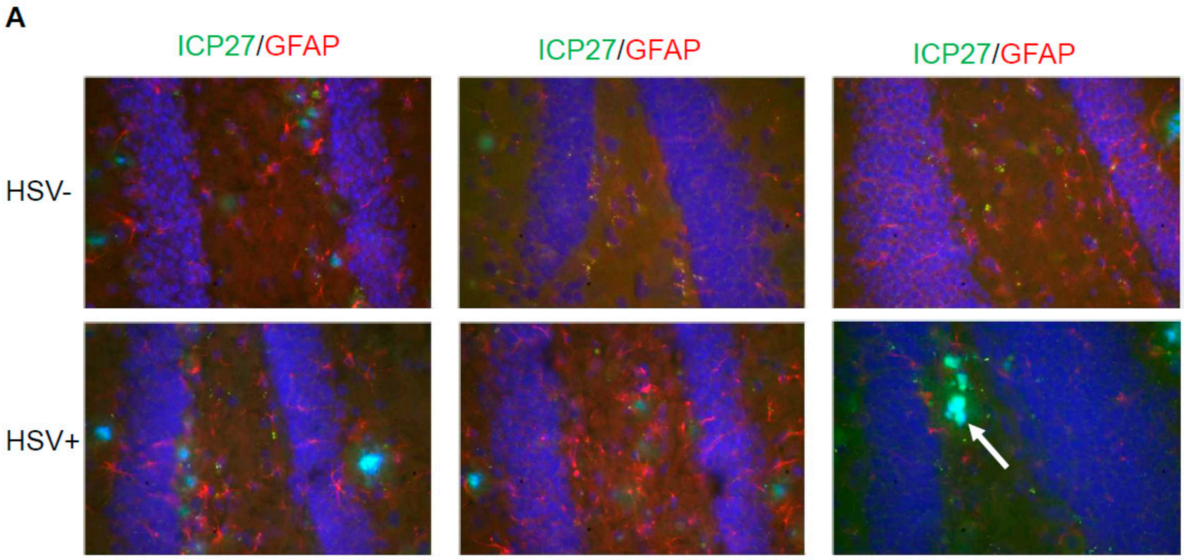

B

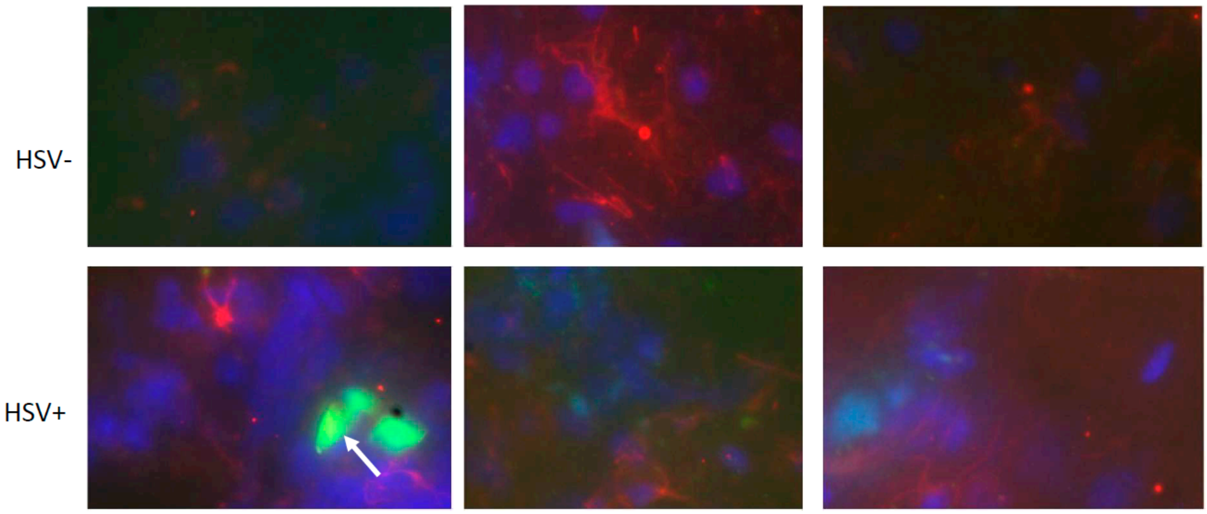

Sup Figure S6. Indirect immunofluorescence analyses of a mouse brain section from Het mice. The sectioned brain was fixed, permeabilized in acetone first, then stained with 1:200 HSV-1 ICP27 monoclonal antibody and 1:2000 Glial Fibrillary Acidic protein (GFAP) rabbit polyclonal antibody first, then a secondary goat anti-mouse antibody conjugated with FITC (Green) and goat anti-rabbit IgG antibody conjugated with Tax red (Red) at a 1:2000 dilution, respectively. **A):** ICP27 and GFAP immunofluorescent staining in DG regions from three different Het/HSV+ and Het/HSV- mice. **B):** ICP27 and GFAP immunofluorescence staining in Cornu Ammonis 1 region (CA1) from three different Het/HSV+ and Het/HSV- mice. Cell nuclei were visualized with DAPI (blue). The images were taken at 40X magnification.

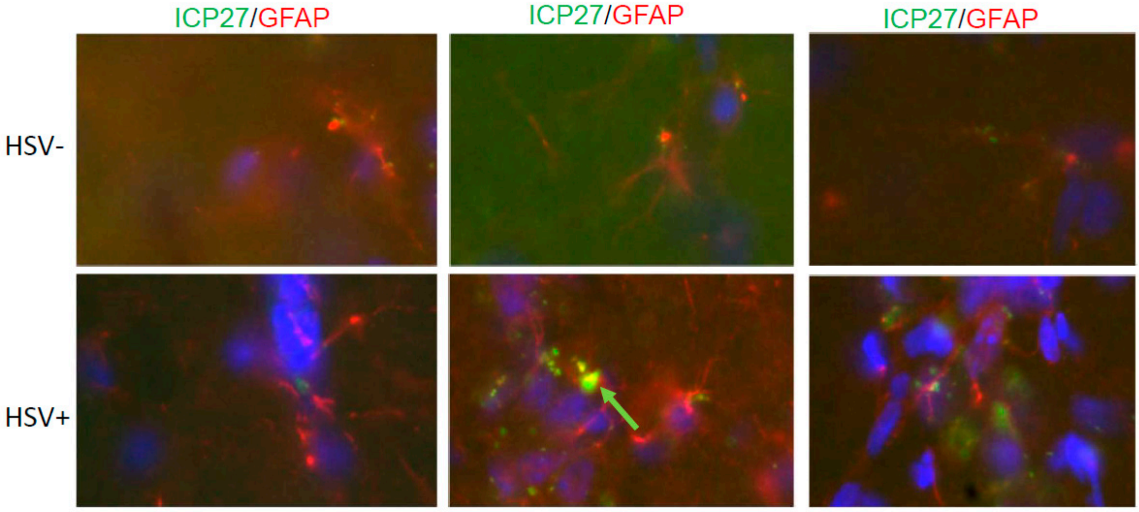

Sup Figure S7. Indirect immunofluorescence analyses of a mouse brain section from WT mice. The sectioned brain stained similarly as in Fig. Sup Fig. 4. Cell nuclei were visualized with DAPI (blue). The images were taken from the Cornu Ammonis 1 (CA1) region of 3 independent mice at 40X. HSV-: uninfected mice; HSV+: infected mice.

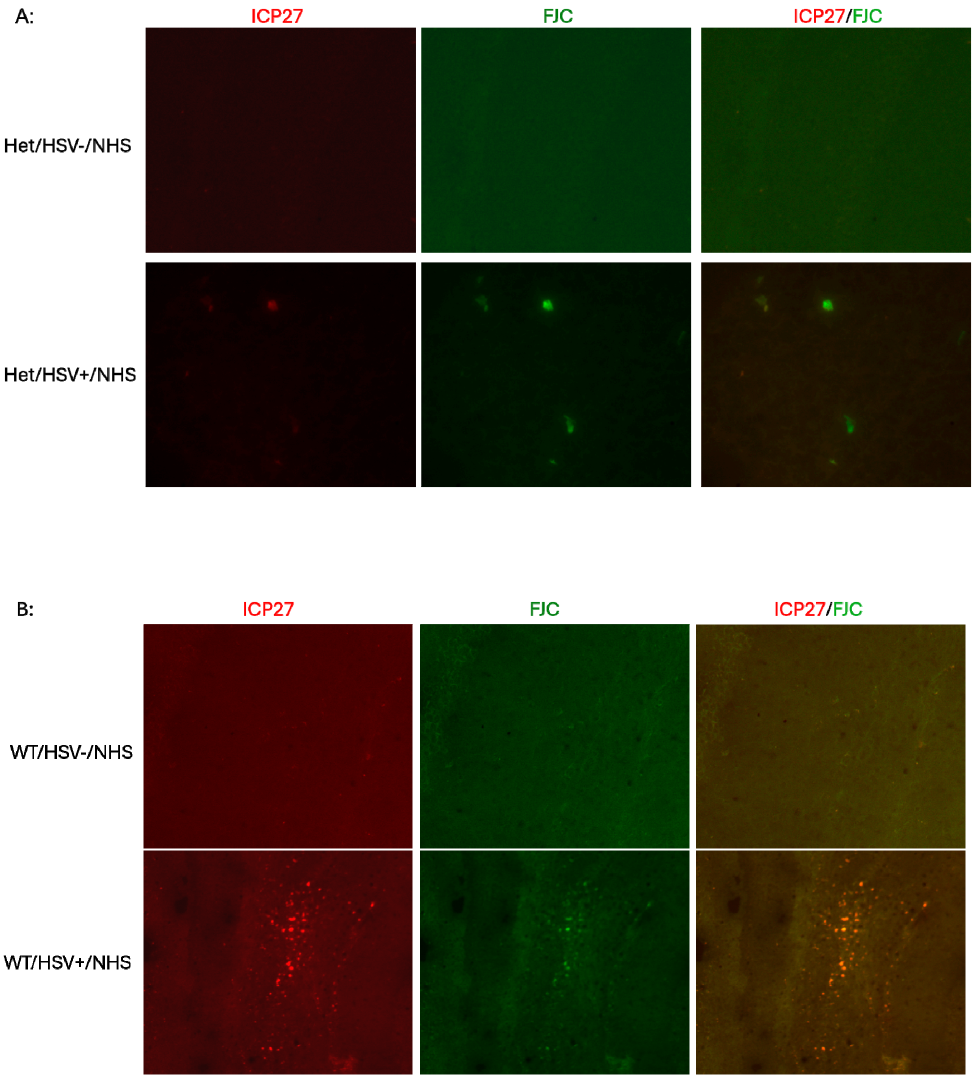

Sup Figure S8. FJC-ICP27 co-staining brain sections from Het and WT mice. The sectioned brain was stained first with FJC (Green) according to the manufacturer's instructions, then fixed in 4% paraformaldehyde, permeabilized in 0.1% TritonX-100, then stained with a 1:200 HSV-1 ICP27 monoclonal antibody first, then a secondary goat anti-mouse antibody conjugated with Tax Red (Red) at a 1:2000 dilution, respectively. **A):** FJC-ICP27 co-staining in Cornu Ammonis 1 region (CA1) from Het/HSV+/NHS and Het/HSV-/NHS mice, respectively. **B)** FJC-ICP27 co-staining in

the Cornu Ammonis 1 region (CA1) from WT/HSV+/NHS and WT/HSV-/NHS mice, respectively. The images were taken at 20X magnification.

Supplementary Table S1. Animal details in behavior analysis tested 3 days post heat stress

**animal ID**	**genotype**	**infection**	**30 dpi**	**60 dpi**	**D3 post-behavior test**
224F1	HET	HSV-1	HS1	HS2	Yes
224F2	HET	HSV-1	HS1	HS2	Yes
224F3	HET	HSV-1	HS1	HS2	Yes
224M2	HET	HSV-1	HS1	HS2	Yes
224M4	HET	HSV-1	HS1	HS2	Yes
227M4	HET	HSV-1	NHS	NHS	Yes
227M5	HET	HSV-1	NHS	NHS	Yes
227M6	HET	HSV-1	NHS	NHS	Yes
220F6	HET	Neg	NHS	NHS	Yes
220F8	HET	Neg	NHS	NHS	Yes
227M1	HET	Neg	NHS	NHS	Yes
228M1	HET	Neg	NHS	NHS	Yes
230M1	HET	Neg	NHS	NHS	Yes
230M2	HET	Neg	NHS	NHS	Yes
224F4	HET	Neg	HS1	HS2	Yes
224F5	HET	Neg	HS1	HS2	Yes
224F8	HET	Neg	HS1	HS2	Yes
224F6	WT	HSV-1	HS1	HS2	Yes
224F7	WT	HSV-1	HS1	HS2	Yes
225F1	WT	HSV-1	HS1	HS2	Yes
224M1	WT	HSV-1	NHS	NHS	Yes
224M3	WT	HSV-1	NHS	NHS	Yes
224M5	WT	HSV-1	NHS	NHS	Yes
228M3	WT	HSV-1	NHS	NHS	Yes
229F1	WT	HSV-1	NHS	NHS	Yes
229F2	WT	HSV-1	NHS	NHS	Yes
222M5	WT	HSV-1	HS1	HS2	Yes
225M3	WT	HSV-1	HS1	HS2	Yes
225M4	WT	HSV-1	HS1	HS2	Yes
222M3	WT	Neg	HS1	HS2	Yes
222M4	WT	Neg	HS1	HS2	Yes
222M5	WT	Neg	HS1	HS2	Yes

222M3	WT	Neg	NHS	NHS	Yes
222M4	WT	Neg	NHS	NHS	Yes
222M5	WT	Neg	NHS	NHS	Yes
225F2	WT	Neg	NHS	NHS	Yes
227F1	WT	Neg	NHS	NHS	Yes
227F2	WT	Neg	NHS	NHS	Yes

Note: M: male; F: female

Supplementary Table S2. Animal details in behavior analysis tested 2 weeks after heat stress

animal ID	genotype	Infection	30 dpi	45 dpi	60 dpi	D15 post-behavior test
298M5	HET	Neg	NHS	NHS	NHS	Yes
302F5	HET	Neg	NHS	NHS	NHS	Yes
308M5	HET	Neg	NHS	NHS	NHS	Yes
298M1	HET	Neg	HS1			Yes
303F1	HET	Neg	HS1			Yes
304F9	HET	Neg	HS1			Yes
297F1	HET	Neg		HS2		Yes
304F7	HET	Neg		HS2		Yes
310M7	HET	Neg		HS2		Yes
298M4-M7	HET	Neg			HS3	Yes
302M1	HET	Neg			HS3	Yes
310M3	HET	Neg			HS3	Yes
310M5	HET	Neg			HS3	Yes
300M4	HET	HSV-1	NHS	NHS	NHS	Yes
304F10	HET	HSV-1	NHS	NHS	NHS	Yes
312M2	HET	HSV-1	NHS	NHS	NHS	Yes
310F4	HET	HSV-1	NHS	NHS	NHS	Yes
296M1	HET	HSV-1	HS1			Yes
311F3	HET	HSV-1	HS1			Yes
302F7	HET	HSV-1	HS1			Yes
304F8	HET	HSV-1	HS1			Yes
296F4	HET	HSV-1		HS2		Yes
303F2	HET	HSV-1		HS2		Yes
304F6	HET	HSV-1		HS2		Yes
300F2	HET	HSV-1			HS3	Yes
304M1	HET	HSV-1			HS3	Yes
308F2	HET	HSV-1			HS3	Yes
296F6	WT	Neg	NHS	NHS	NHS	Yes
298M6	WT	Neg	NHS	NHS	NHS	Yes

299M1	WT	Neg	NHS	NHS	NHS	Yes
304M3	WT	Neg	NHS	NHS	NHS	Yes
302F6	WT	Neg	NHS	NHS	NHS	Yes
303F3	WT	Neg	NHS	NHS	NHS	Yes
310F2	WT	Neg	NHS	NHS	NHS	Yes
310F3	WT	Neg	NHS	NHS	NHS	Yes
296F5	WT	Neg			HS3	Yes
298F3	WT	Neg			HS3	Yes
299F10	WT	Neg			HS3	Yes
302M2	WT	Neg			HS3	Yes
302M3	WT	Neg			HS3	Yes
299F7	WT	HSV-1	NHS	NHS	NHS	Yes
299F9	WT	HSV-1	NHS	NHS	NHS	Yes
296M2	WT	HSV-1	NHS	NHS	NHS	Yes
296M3	WT	HSV-1	NHS	NHS	NHS	Yes
300M5	WT	HSV-1	NHS	NHS	NHS	Yes
312M3	WT	HSV-1	NHS	NHS	NHS	Yes
297M2	WT	HSV-1		HS2		Yes
297M4	WT	HSV-1			HS3	Yes
299M3	WT	HSV-1			HS3	Yes
300F1	WT	HSV-1			HS3	Yes
304M2	WT	HSV-1			HS3	Yes
310F1	WT	HSV-1			HS3	Yes

Note: F: female; M: Male; NHS: no heat stress; HS1: stressed once on 30 dpi; HS2: stressed twice on 30 dpi and 45 dpi, respectively; HS3: stressed three times on 30 dpi,
